# Progressive transfer learning for advancing machine learning-based reduced-order modeling

**DOI:** 10.1038/s41598-024-64778-y

**Published:** 2024-07-08

**Authors:** Teeratorn Kadeethum, Daniel O’Malley, Youngsoo Choi, Hari S. Viswanathan, Hongkyu Yoon

**Affiliations:** 1 Sandia National Laboratories, Albuquerque, NM 87185 USA; 2 Los Alamos National Laboratory, Los Alamos, NM 87545 USA; 3 Lawrence Livermore National Laboratory, Livermore, CA 94550 USA

**Keywords:** Hydrology, Solid Earth sciences

## Abstract

To maximize knowledge transfer and improve the data requirement for data-driven machine learning (ML) modeling, a progressive transfer learning for reduced-order modeling (p-ROM) framework is proposed. A key concept of p-ROM is to selectively transfer knowledge from previously trained ML models and effectively develop a new ML model(s) for unseen tasks by optimizing information gates in hidden layers. The p-ROM framework is designed to work with any type of data-driven ROMs. For demonstration purposes, we evaluate the p-ROM with specific Barlow Twins ROMs (p-BT-ROMs) to highlight how progress learning can apply to multiple topological and physical problems with an emphasis on a small training set regime. The proposed p-BT-ROM framework has been tested using multiple examples, including transport, flow, and solid mechanics, to illustrate the importance of progressive knowledge transfer and its impact on model accuracy with reduced training samples. In both similar and different topologies, p-BT-ROM achieves improved model accuracy with much less training data. For instance, p-BT-ROM with four-parent (i.e., pre-trained models) outperforms the no-parent counterpart trained on data nine times larger. The p-ROM framework is poised to significantly enhance the capabilities of ML-based ROM approaches for scientific and engineering applications by mitigating data scarcity through progressively transferring knowledge.

## Introduction

For achieving net-zero emissions and sustainable societal growth, managing and optimizing underground resources is very important to produce energy sustainably, extract groundwater responsibly, store undesirable substances underground, and many other activities^1–3^. However, understanding the complex multiphysics phenomena that govern subsurface physics and characterize underground structures poses significant challenges. These structures are often anisotropic, heterogeneous, and exhibit discontinuous material properties that span multiple orders of magnitude^4–8^. Laboratory experiments are critical to enhancing our understanding of natural and engineering physical, chemical and biological processes in these complex systems^9–12^, but limited in experimental conditions^13^. On the other hand, field experiments and real-field operations, which are often expensive and time-consuming, are difficult to control and observe the consequences accurately^14^. To overcome the limits in these actual experiments, high-fidelity computational modeling are utilized to generalize our prediction capability of coupled physical, chemical, and biological processes across different scales under varying conditions^15^. However, their use often entails a high computational cost^16–18^, resulting in prohibitively expensive computational needs for optimization and uncertainty quantification^19,20^.

Reduced-order modeling (ROM) based on Machine Learning (ML) offers computationally efficient solutions for spatio-temporal physical problems^21–23^. Even though this approach accelerates large-scale inverse modeling, optimizations, and uncertain quantification processes^24–26^, it also requires a substantial amount of training samples. To address the challenge of data scarcity in ML-based ROM, efficient learning methods like transfer learning^27,28^ and progressive learning^29,30^ can be employed.

Transfer learning involves leveraging pre-trained models to enhance the current model’s performance^27^. Commonly used techniques in transfer learning include fine-tuning and adding fresh layers. Fine-tuning involves utilizing the entire set of trainable parameters from a pre-trained model with a small learning rate. This allows the model to adapt to a new task while retaining knowledge from previous training, which is particularly useful when the pre-trained model’s features are relevant to the current task. On the other hand, adding fresh layers involves using pre-trained trainable parameters with new layers added or specific layers replaced. This method is beneficial when the pre-trained model captures generic features that serve as a starting point for the current task. Recent advancements in transfer learning also involve using Kernel methods, such as neural tangent kernels, to enhance the generalization and scalability of the process^31^.

Although transfer learning techniques offer significant benefits, they also possess three notable drawbacks. First, current procedures only leverage knowledge from one model and apply it to the next (i.e., one-to-one transfer)^28^ rather than learning from multiple trained models. Second, there is a risk of catastrophic forgetting in which the newly trained model completely loses the knowledge of the previous task and cannot be applied without retraining^29^. Lastly, understanding the factors contributing to the success or failure of transfer learning algorithms can be challenging. It is often complex to determine when and why certain algorithms work and identify which information enhances the transfer process, as highlighted in recent studies^31,32^. These limitations highlight the need for learning approaches that enable selective knowledge transfer while preserving previously acquired information. The survey by Zhuang et al.^28^ provides a valuable resource for a comprehensive understanding of transfer learning techniques, including their advantages and disadvantages.

**Figure 1:**
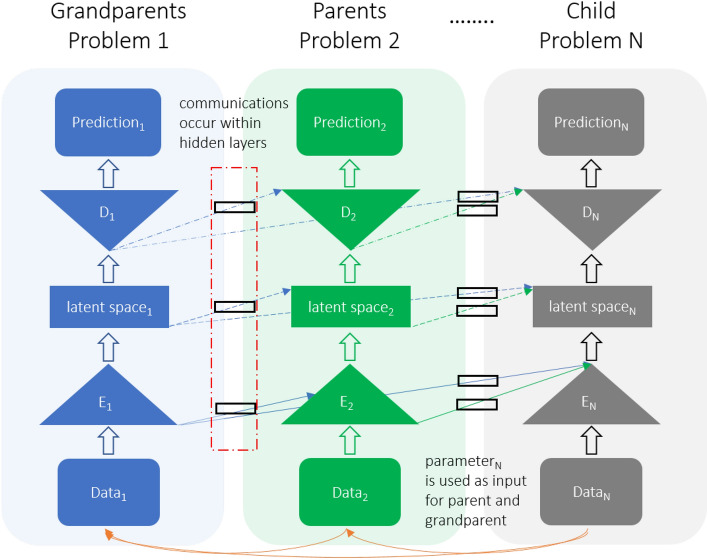
Schematic of multi-level progressive transfer learning. $N-1$ pre-trained ML models for $N-1$ different problems can accelerate training and improve accuracy for a new ML model addressing a new problem. E and D represent the Encoder and Decoder, respectively. The black rectangles denote combined trainable parameters for the child model. During training/inference, the input of the child model, $\textrm{Data}_{\textrm{N}}$, is also used as input for its parent (Problem 2) and grandparent (Problem 1) models. If the input dimensions differ between parent and child models, a single linear layer (adapter) addresses the discrepancy. The parameters of Problems 1 and 2 are frozen during the child’s training, while any adapters must be trained concurrently with the child model.

Progressive neural networks (PNN) have been developed to address the challenge of many-to-one transfer and mitigate the issue of catastrophic forgetting, leveraging prior knowledge from previously trained models through their lateral connections^29^. PNN is often associated with lifelong or continual learning, where a model continues learning from new data and tasks without forgetting previously learned information^30^. Hence, PNN or progressive learning provides an efficient knowledge transfer mechanism that allows us to utilize multiple models for spatio-temporal problems (e.g.,^33–35^) while reducing burdens of generating big training data. In this work, progressive reduced-order models (p-ROM), built upon the PNN concept, aim to capitalize on prior knowledge and make the most of previously developed models; see Fig. 1. Compared with ongoing research on transfer learning, this framework progressively accumulates common features of physics of interest and expands them instead of fine-tuning (including adding fresh layers)^27,28^ or enforcing loss functions^36^, resulting in immunization to catastrophic forgetting. Besides, it can transfer learning from many trained models instead of one-to-one.

The main ingredient of this framework lies within gates used to control information flow from previous generations (e.g., grandparents and parents)^29,37^. These gates are pivotal in controlling information flow while concurrently training a child model. The total trainable parameters consist of a combination of trainable parameters from the child model and trainable parameters from all the gates associated with the child model, as indicated by the black squares in Fig. 1. Each gate corresponds to a connection between the child and a preceding model (grandparents or parents in this illustration). During the training process, if the information from a prior model proves beneficial (i.e., it reduces the evaluation of a loss function), the corresponding gate increases the flow of information and vice versa. In this context, we employ a linear layer as a gate, meaning that the trainable parameters of each gate are adjusted in response to evaluating the loss function. It’s worth noting that one can enhance the complexity of these gates by employing techniques such as recurrent units^38^, attention mechanisms^39^, or adaptive algorithms^40^. It is also noted that there are other PNN approaches, such as one that employs three procedures: curriculum, progression, and pruning^30^. The curriculum procedure actively selects tasks from a set of candidates, progression expands the model’s capacity by adding new parameters that leverage prior knowledge, and pruning mitigates parameter growth and negative interference from unrelated prior knowledge.

Since this framework, as shown in Fig. 1 is non-intrusive or data-driven in nature (i.e., it does not require a knowledge of underlying physics, governing equations, or modifications inside high-fidelity software), it enables our proposed platform to learn from physics simulators that can be treated as black-box. Our framework allows for defining parent and child models based on entirely distinct problems, each with its unique input parameters. For example, a child model might focus on solid deformation, with boundary conditions as its parameters. In contrast, a parent model might address fluid flow, utilizing a Rayleigh number as its input parameter. This versatility is attributed to our framework’s design, where the communication between each problem—whether parent or child—occurs within the hidden layers. This design choice ensures that the interactions are not limited by the specifics of the input (data or parameters) or the output (predictions) layers, as illustrated in Fig. 1. Throughout this study, we will use the Barlow Twins ROM (BT-ROM)^34^ to evaluate our p-ROM framework (p-BT-ROM hereafter). BT-ROM represents a cohesive framework harnessing self-supervised embedding learning. It demonstrates remarkable performance across linear and nonlinear domains, serving as a versatile and adaptable solution capable of seamless operation on unstructured meshes. This quality facilitates its smooth integration with conventional numerical solvers and real-field measurements. Note that this framework can be integrated into any data-driven ML-based ROMs.

This work will test a series of physics-based problems ranging from transport in porous media and gravity-driven flow to a finite deformation of a hyperelastic material to emphasize that transferring knowledge among different physics (fluid to solid mechanics, in this case) is possible. Besides, we employ a range of topologies from 2- to 3-Dimensional domains to further emphasize the possibilities of transferring knowledge across different topologies. Our work aims to demonstrate that the proposed framework effectively mitigates the data scarcity challenge, often associated with approaches like few-shot learning^41^, within data-driven methodologies. Instead of starting anew for each task, we systematically build upon existing models, harnessing the knowledge and insights acquired from prior models to inform and enhance subsequent iterations. This strategy conserves time and resources and significantly improves the overall efficiency of data-driven ML.

## Results

In this section, we present the proposed progressive learning framework to demonstrate enhanced accuracy compared to models lacking prior knowledge (without a parent) and achieve comparable or superior accuracy to models trained on larger datasets (more training samples but without a parent). We assess the performance of the progressive Barlow Twins reduced-order model (p-BT-ROM) by evaluating its results on a range of physics problems discussed in Sections Data generation and Supplementary sec. 2.

### Data generation

Four physics problems presented in Table 1 are used for performance evaluation. The first problem, referred to as Problem #1, involves the study of transport in porous media with varying velocity fields as the parameter space and the concentration as the quantity of interest. For Problem #2, transport scenarios with varying diffusivity coefficients are examined with the diffusivity coefficient as the parameter space and the concentration as the quantity of interest. For problem #3 adapted from Kadeethum et al.^42^, gravity-driven flow in porous media is used with the Rayleigh number ($\textrm{Ra}$) as the parameter and fluid temperature as the quantity of interest. Finally, Problem #4 addresses the finite deformation of a hyperelastic material with the displacement at a boundary and the displacement within the source term as the parameter space in 2-Dimensional (2-D) and 3-D cases, respectively. In both cases, our primary interest lies in the magnitude of solid displacement. This problem is adapted from the work of Kadeethum et al.^43^. For comprehensive details regarding the problem settings, quantities of interest, parameter space, and the methodology for generating our training, validation, and testing datasets, please refer to Supplementary sec. 4.

**Table 1:** Summary of physical problems.

Problem	Dimension	Physics	State variables	Quantity of interest	Parameters	Section
#1	2	Transport in porous media	Concentration	Concentration	Fluid velocity	Supplementary sec. 2.1
#2	2	Transport in porous media	Concentration	Concentration	Diffusivity coefficient	Supplementary sec. 2.2
#3	2	Gravity-driven flow	Pressure	Temperature	Rayleigh number	Supplementary sec. 2.3
			Velocity			
			Temperature			
#4	2	Solid deformation	Displacement	Magnitude of displacement	Boundary condition	Supplementary sec. 2.4
	3				Source term	

### Investigation strategy

In the Results section, we have designed two numerical experiments to address how the p-ROM framework can be applied to different physics and topologies.

1. First experiment: knowledge transfer between physics with fixed topology
  Objective: Determine if the progressive learning framework can transfer knowledge between different physics problems while maintaining a fixed topology (i.e., same mesh and connectivity).
  Parent Problems (Fluid): Problems #1, #2, and #3
  Child Problem (Solid): Problem #4
  Implication: Successful knowledge transfer implies the framework’s ability to share knowledge across different physics domains.
2. Second experiment: knowledge sharing between different topologies in multiple physics
  Objective: Investigate whether the progressive learning framework can share knowledge between different topologies.
  Parent Problems (2-D Topology): Problems #1, #2, #3, and #4
  Child Problem (3-D Topology): Problem #4
  Implication: Successful knowledge sharing indicates the framework’s potential to transfer knowledge across different dimensions and topologies.

In the second experiment, we highlight our Problem #4, where solid mechanics with deformation to the external forces are solved with multiple parents with different physics and topologies. This case demonstrates the versatility and adaptability of the p-ROM in integrating complex, domain-specific knowledge from both fluid and solid mechanics. This approach highlights the potential for such systems to transcend traditional disciplinary boundaries, offering insights into the transferability of learning processes across distinct scientific landscapes.

For a systematic evaluation of the proposed approach, Stage 1 with a similar topology (see Supplementary sec. 5.5) is presented first, followed by Stage 2 with different topologies. All results are summarized in Table 2. Overall, two key outcomes are highlighted. First, more parents result in better model accuracy, with knowledge transfer working across different physics and topologies, from fluid to solid mechanics and 2-D to 3-D domains. Second, the p-ROM tackles data-hungry issues. For example, a 4-parent model in the different topology problems outperforms a model without the parent, even when the latter is trained with nine times more data. In short, tapping into existing knowledge of previously trained models using the p-ROM framework improves accuracy and data requirements. It facilitates knowledge transfer across domains and topologies, making for a less data-dependent, data-driven model. We will provide detailed analyses in the following sections.

**Table 2:** Summary of results for test sets.

Setting		Num. parent(s)	Training samples	Avg.MAE	Std.MAE	Remark
Similar topology	Problem #3	0	$\textrm{M} = 40$ ($\textrm{M}N_t = 28966$)	2.41E-03	1.27E-03	Large training set
		0	$\textrm{M} = 5$ ($\textrm{M}N_t = 3623$)	1.61E-02	5.83E-03	Small training set
		1	$\textrm{M} = 5$ ($\textrm{M}N_t = 3623$)	5.87E-03	2.75E-03	From transport to gravity-driven problems
		2	$\textrm{M} = 5$ ($\textrm{M}N_t = 3623$)	4.82E-03	2.36E-03	
	Problem #4	0	$\textrm{M} = 1600$	1.08E-04	4.84E-05	Large training set
		0	$\textrm{M} = 100$	3.69E-03	1.93E-03	Small training set
		1	$\textrm{M} = 100$	3.79E-04	7.19E-05	From fluid to solid problems
		2	$\textrm{M} = 100$	3.70E-04	6.52E-05	
		3	$\textrm{M} = 100$	3.20E-04	4.40E-05	
Different topologies	Problem #2	0	$\textrm{M} = 20$ ($\textrm{M}N_t = 9620$)	3.99E-03	1.64E-03	Large training set
		0	$\textrm{M} = 3$ ( $\textrm{M}N_t = 1443$)	2.68E-02	7.77E-03	Small training set
		4	$\textrm{M} = 3$ ( $\textrm{M}N_t = 1443$)	4.01E-03	2.48E-03	From 1 to 2 obstacles
	Problem #3	0	$\textrm{M} = 20$ ($\textrm{M}N_t = 10832$)	9.77E-03	5.25E-03	Large training set
		0	$\textrm{M} = 5$ ($\textrm{M}N_t = 2706$)	4.06E-02	1.32E-02	Small training set
		4	$\textrm{M} = 5$ ($\textrm{M}N_t = 2706$)	3.84E-03	2.16E-03	From 1 to 3 holes obstacles
	Problem #4	0	$\textrm{M} = 1600$	2.64E-03	1.73E-03	Large training set
		0	$\textrm{M} = 100$	6.66E-03	3.17E-03	Small training set
		4	$\textrm{M} = 100$	8.79E-04	3.61E-04	From 2- to 3-D topologies

Child$\sim$parent(s) relationship can be found in Supplementary figs. 12 and 14 for the similar topology cases, and Fig. 4 for different topologies cases.

### Similar topology

**Figure 2:**
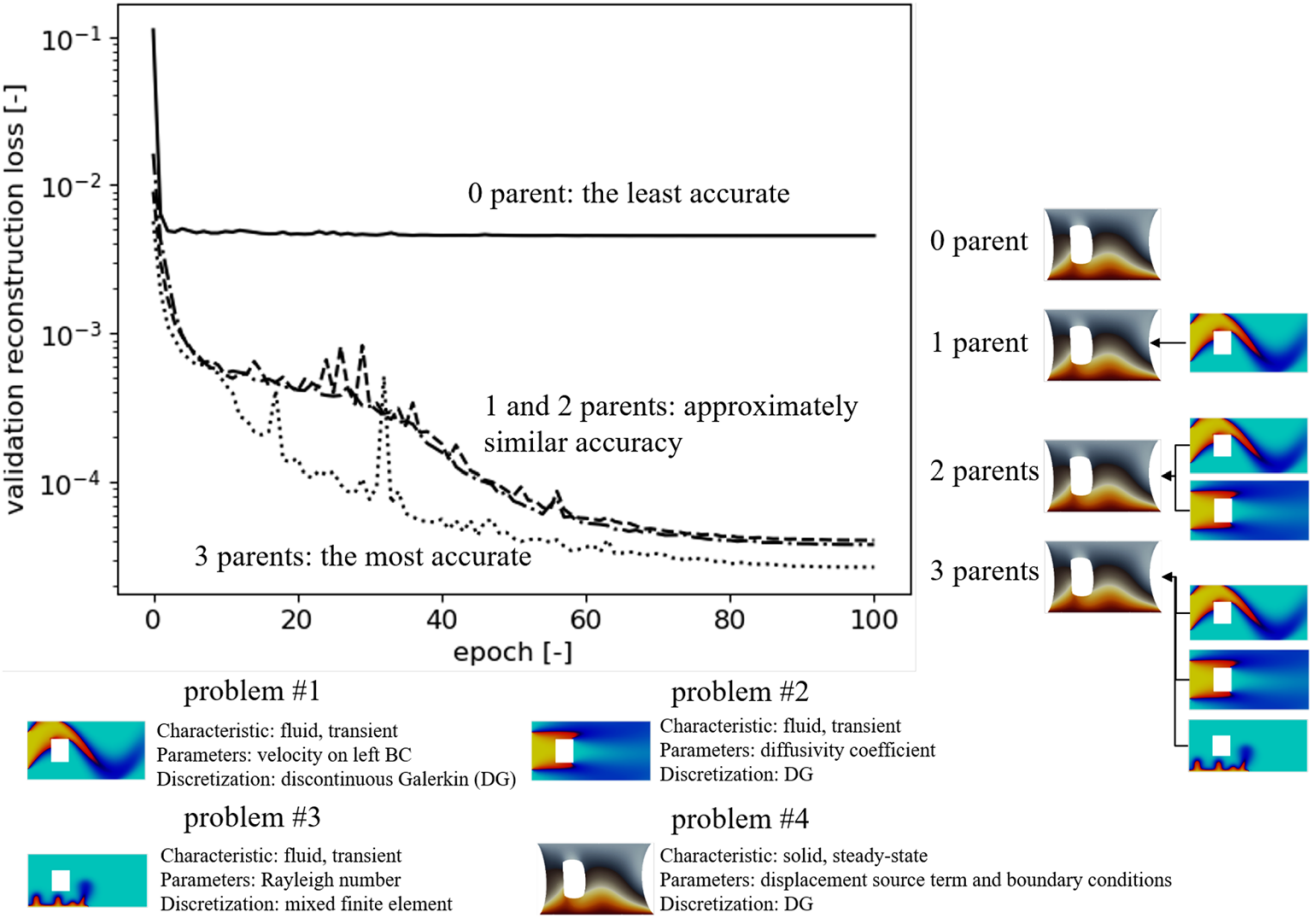
Illustration of the proposed framework to transfer knowledge from fluid mechanics (Problems #1-#3) to solid mechanics (Problem #4) problems using the reconstruction loss of the validation set for Problem #4 in a 2-D domain. This loss reflects the training phase of the (p-)BT-ROM (see Supplementary fig. 2—third step). Note that 0 parents for p-BT-ROM represent the original BT-ROM, and for Problem #4, the results in the deformed configuration are shown.

Here, we intend to illustrate that progressive learning enables us to transfer knowledge to problems with different underlying physics (e.g., fluid to solid mechanics) and parameter spaces (e.g., boundary conditions or material properties), see Fig. 2 and Table 2 for a summary of results for validation and test sets, respectively. In Fig. 2 reconstruction loss (Supplementary eq. 14) results of the validation set (as a function of the number of epochs and parents) are presented using Problem #4 in a 2-D domain to introduce our framework’s benefit for the training phase of the (p-)BT-ROM (see Supplementary fig. 2—third step). The detail of the problem geometry is shown in Supplementary fig. 1a, and more comprehensive results can be found in Supplementary sec. 5.3. Our approach involves a combination of p-BT-ROM, utilizing the weights and biases of the trained models as an initialization. In-depth results and rationale for adopting this approach are presented from Supplementary sec. 5.1 to Supplementary sec. 5.4. Fig. 2 clearly shows that the p-BT-ROM model’s performance increases with increasing the number of parents (i.e., more pre-trained models). These results imply that the p-BT-ROM could transfer knowledge from fluid problems (Problems #1 to #3) to a solid problem (Problem #4). It should be noted that trainable parameters inevitably become larger as more parents are utilized (see Supplementary sec. 6). These additional parameters lead to a heavier computational time and memory load, but the computational cost still remains much lower than the physical model.

For the testing process, the p-BT-ROM model was trained with a specific number of epochs (100 in this case) with the training set (see Supplementary fig. 2—third step). Subsequently, a set of weights and biases was selected to deliver the lowest loss for a validation set (still Supplementary fig. 2—third step). Finally, the decoder of p-BT-ROM is employed with the set of weights and biases in combination with radial basis function (RBF) interpolator for the evaluation using a test set (see Supplementary fig. 2—fourth and fifth steps). Overall, the test sets’ results in Supplementary sec. 5.5 show that p-BT-ROM’s accuracy increases with increasing the number of parents, which is similar to the behaviors in the validation cases (Fig. 2). As expected, the BT-ROM (0 parent)^34^ has the worst accuracy. However, even the model with many parents still could not achieve the same level of accuracy as the model that was trained with a very large training set with 0 parent (BT-ROM). This behavior implies the benefits of adding training samples that PNNs might be unable to compensate for or substitute. On the other hand, they can produce more accurate results for fixed-sized datasets (supplemented with data from the simpler parent problems).

### Different topologies

**Figure 3:**
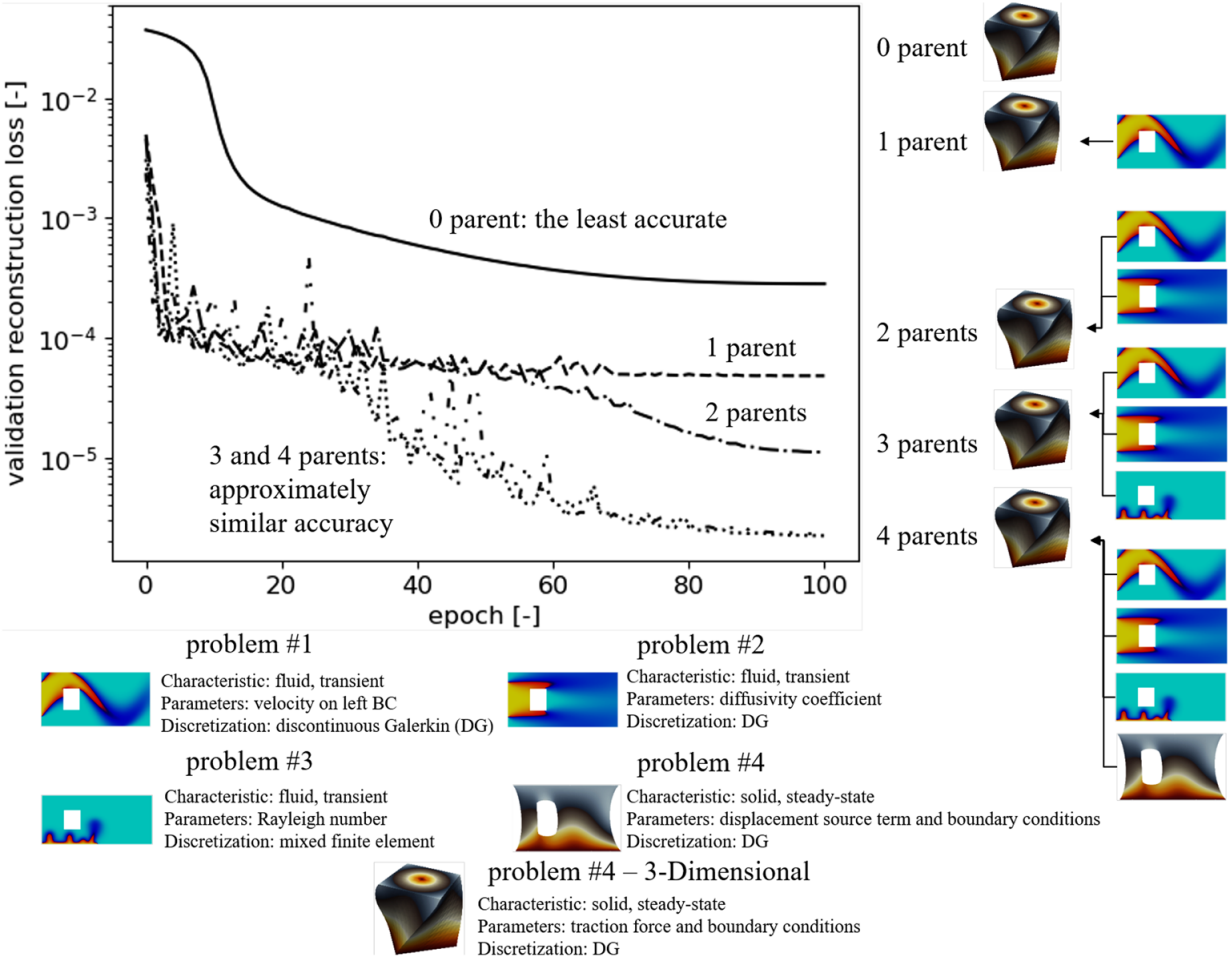
Illustration of the proposed framework to transfer knowledge from 2-D to 3-D problems using the reconstruction loss of the validation set for Problem #4 in a 3-D domain. This loss reflects the training phase of the (p-)BT-ROM (see Supplementary fig. 2 - third step). Note that for Problem #4 we show the results in the deformed configuration.

In this study, we highlight the efficacy of the p-ROM approach in knowledge transfer across various topologies, including shifts from 2-D to 3-D domains and variations in 2-D domains with multiple obstacles. The validation reconstruction loss (Supplementary eq. 14) of different numbers of parents for Problem #4 in a 3-D domain is illustrated in Fig. 3 (and also Supplementary fig. 1d). Like the previous section, the p-BT-ROM model enhances its accuracy with an increase in the number of parents, with minimal performance discrepancy between 3 and 4 parents. These findings affirm the viability of knowledge transfer across diverse topologies and spatial dimensions, ultimately improving the precision of the data-driven framework. However, it’s worth noting that, akin to similar topology scenarios, employing more parents expands the trainable parameter space, increasing training costs. For a detailed parameter assessment, please refer to Supplementary sec. 6.

**Figure 4:**
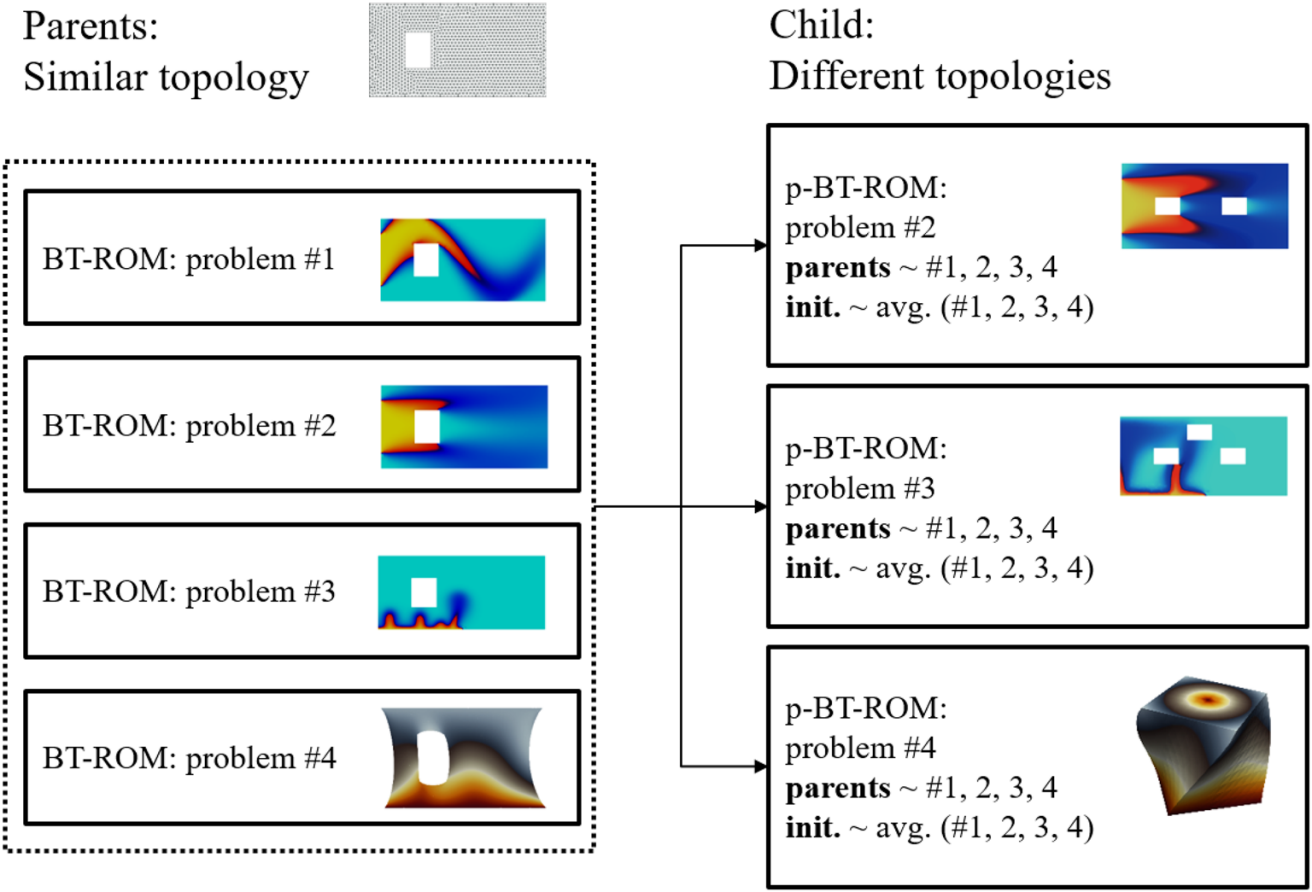
Different topologies - schematic of p-BT-ROM specifies child−parent(s) relationship. See Supplementary fig. 1 for more information on the geometries.

Next, the p-BT-ROM’s performance on the test set is evaluated. The goal here is to assess the p-BT-ROM’s capability to improve accuracy while limited training samples are provided. Fig. 4 shows the schematic of child−parent(s) relationships evaluated with the p-BT-ROM using the small dataset from Supplementary sec. 5.5.2. For the four parents BT-ROM models, we use four different problem sets, including Problem #1 with a training set of $\textrm{M}N_t = 2405$ ($\textrm{M} = 5$), Problem #2 with $\textrm{M}N_t = 2405$ ($\textrm{M} = 5$), Problem #3 with $\textrm{M}N_t = 3623$ ($\textrm{M} = 5$), and Problem #4 with $\textrm{M} = 100$ (see also Table 2). These cases represent realistic scenarios where only a few training samples for complex problems are available, but larger datasets are readily available for more straightforward problems. This setting also illustrates that our framework can accommodate and combine different types of physics problems (i.e., fluid and solid mechanics problems) and temporal characteristics (i.e., transient and steady-state problems) into the list of parent models.

**Figure 5:**
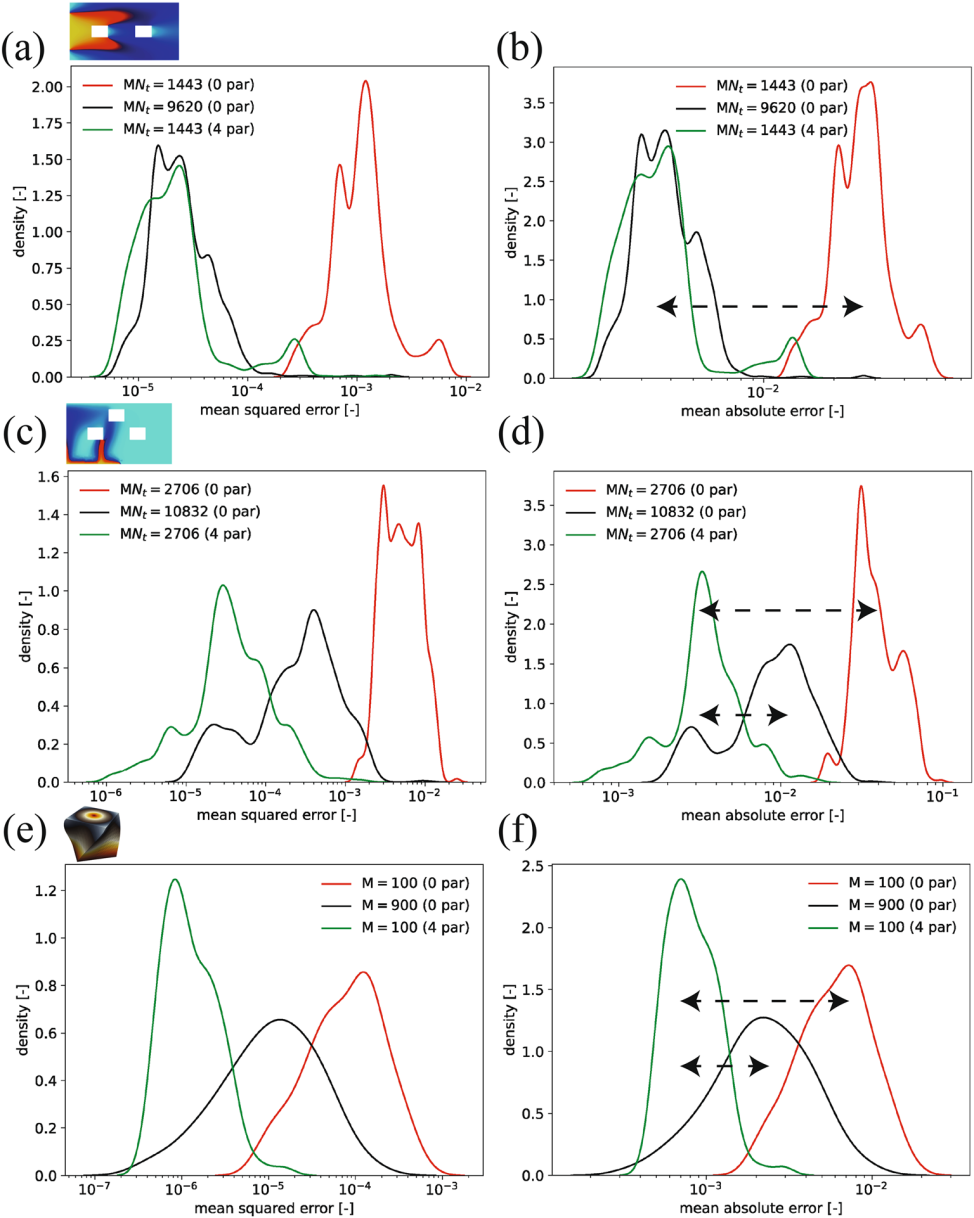
Performance comparison of p-BT-ROM (4 parents) with BT-ROM (0 parent) in (**a**, **c**, **e**) mean squared error (MSE, Supplementary eq. 19) and (**b**, **d**, **f**) mean absolute error (MAE, Supplementary eq. 20) using a different number of training data ($\textrm{M}N_t$ or $\textrm{M}$). A snapshot of the solution (Supplementary fig. 4) is also inserted for each Problem. (a,b) Problem #2 (topology in Supplementary fig. 1b) with $\textrm{M}N_t = 1443$ ($\textrm{M} = 3$) and $\textrm{M}N_t = 9620$ ($\textrm{M} = 20$), (c,d) Problem #3 (topology in Supplementary fig. 1c) with $\textrm{M}N_t = 2706$ ($\textrm{M} = 5$) and $\textrm{M}N_t = 10832$ ($\textrm{M} = 20$), and (e,f) Problem #4 (topology in Supplementary fig. 1d) with ($\textrm{M} = 100$) for 0 parent (red) and 4 parents (green) cases. In (e,f), solid black lines represent the large training set ($\textrm{M} = 900$). Dashed horizontal black lines show an accuracy gain by including knowledge from parents in p-BT-ROM.

A comparison of p-BT-ROM’s performance with BT-ROM (0 parent) is shown in Fig. 5. A sample snapshot of model prediction for each problem is illustrated in Supplementary fig. 4. For each problem, the p-BT-ROM with 4 parents using the small training set is compared with the BT-ROM (0 parent) using the same small training set and a large training set. For all three problems, the p-BT-ROM performs much better than the BT-ROM (0 parent) with the same number of training sets. However, compared with the BT-ROM with a much larger number of training data, the p-BT-ROM performs similarly for Problem #2 (transport in porous media) while better for the other two Problems #3 and #4 (gravity-driven flow and hyperelasticity). In addition, both mean squared error (MSE) and mean absolute error (MAE) distributions for all three problems are similar, implying that the p-BT-ROM improves the model accuracy for the metric that is not a part of training loss (i.e., MSE is the training loss, but MAE is not).

In addition to these results, comprehensive numerical examples are provided in supplementary sections to enhance the understanding of our work. Specifically, Supplementary sec. 5.1 demonstrates the conventional approach of transfer learning, where pre-trained models’ weights and biases are utilized to initialize a new model (referred to as “init”)^27,28^. This approach serves as a benchmark for comparison with p-BT-ROM, presented in Supplementary sec. 5.2. Furthermore, we explore the potential benefits of combining initialization with p-BT-ROM, denoted as “p-BT-ROM with init.” In Supplementary sec. 5.4, we conclude that the preferred approach going forward is to employ p-BT-ROM with init. To supplement our findings in Section Similar topology, we provide additional results of p-BT-ROM with init for Problems #1, #2, #3, and #4 with fixed topology in Supplementary sec. 5.5. This supplementary section sheds light on the performance of our framework when applied to different problems. Additionally, we have thoroughly investigated the impact of varying the number of parents within our framework to assess its effectiveness, yielding valuable insights.

## Discussion

Through a series of numerical experiments, we demonstrate the effectiveness of a p-ROM framework, which relies on gates to regulate information flow from previously trained models to improve the current model accuracy even when training samples are scarce. These gates are pivotal in managing information flow while concurrently training the current model. The total trainable parameters consist of both current model parameters and those from associated gates. Each gate corresponds to a connection between the current model and a preceding one, adjusting its flow based on the performance impact observed during training. In this context, we employ a linear layer as a gate, which means that the $\textbf{W}$ and $\textbf{b}$ parameters of each gate are adjusted in response to evaluating the loss function. It’s important to note that the complexity of these gates can be enhanced by utilizing techniques such as recurrent units^38^, attention mechanisms^39^, or adaptive algorithms^40^.

Our experiment outcomes, summarized in Table  2, provide compelling evidence that leveraging knowledge from prior models addresses the challenge of limited training data. Notably, the p-ROM, incorporating four parent models, outperforms its counterpart trained with nine times more data. This underscores the potential of utilizing prior knowledge to enhance model performance. Furthermore, our research demonstrates the feasibility of transferring knowledge across diverse physics domains, from fluid to solid mechanics, and various domain topologies, including transitions from 2-D to 3-D domains. However, this benefit comes with an associated computational cost. As the number of parents increases, so does the number of trainable parameters, impacting time and space complexities (Supplementary sec. 6). For our case, training with 0 and 3 parents shows an approximate 10 % increase in wall time using NVIDIA Quadro RTX 8000, while prediction times remain similar (less than a second while running a single high-fidelity model for Problem #3 would typically take at least 30 min). We also note that introducing additional parents into the training process does not merely extend the duration but also escalates the complexity of the training. This is primarily due to the increase in the number of trainable parameters, which, while intended to enhance the model’s learning capability, may inadvertently lead to suboptimal solutions.

The p-ROM concept is versatile and applicable to various machine-learning architectures beyond the p-BT-ROM. It isn’t restricted to specific application domains, such as fluid or solid mechanics, and can adapt to different data sources, including simulations or field measurements. This flexibility allows the framework to accumulate knowledge from diverse sources, enhancing overall performance.

While our study demonstrates the potential of p-ROM, five limitations should be acknowledged. The first one is more complex geometries and topologies should be used to test this framework. Our framework does not have direct information on topologies at this stage. Still, one could use an additional network such as (graph-)trunk nets^44^ to embed the topologies of each parent and child into the framework. Secondly, as mentioned in Supplementary sec. 4 we uniformly sample our parameter space $\varvec{\mu }$; subsequently, one might argue that if we perform a smart sampling or adaptive sampling^45^, we might also end up using less training samples similarly to p-BT-ROM. Hence, one also wants to combine our progressive framework with an adaptive sampling technique to reduce training samples further (or maintain the number of training samples but improve the models’ accuracy).

Thirdly, as the number of parents in the proposed progressive learning framework increases, more trainable parameters are required (Supplementary sec. 6). This results in a more intricate and computationally expensive training process. To address this challenge, pruning can be utilized to effectively maintain the trainable parameters with new child problems while a set of parents’ models can be optimally selected^30,46^. This approach would help optimize the number of trainable parameters, ensuring that only the most helpful knowledge contributes to training the ROM for a specific downstream application, such as $\textrm{CO}_{2}$ storage.

**Figure 6:**
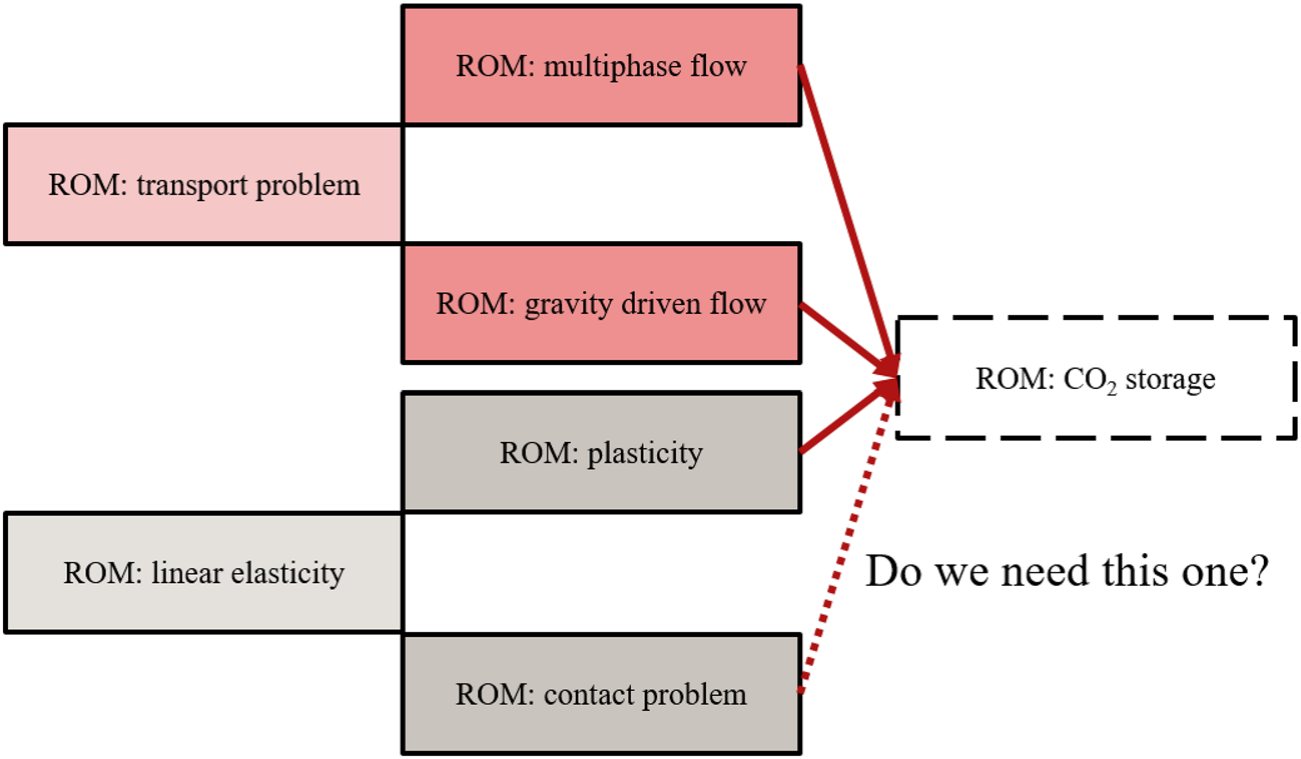
A hypothetical situation where we want to develop a ROM of $\textrm{CO}_{2}$ storage and have trained ROMs of linear elasticity, contact problem, plasticity, transport problem, gravity-driven flow, and multiphase flow in our inventory that we can pick as (grand)parent(s) of $\textrm{CO}_{2}$ storage ROM. We use red to represent fluid problems and grey to show solid problems. Lighter shades represent grandparents, while darker ones are for parents.

For instance, in Fig. 6, we have multiple trained models available in our inventory. In the case of developing a ROM for $\textrm{CO}_{2}$ storage, a straightforward approach would be to utilize all the trained models (i.e., six parents) as parents for the $\textrm{CO}_{2}$ storage ROM. However, considering the increased number of trainable parameters with more parents, a pruning framework can determine which parents are truly influential for the $\textrm{CO}_{2}$ storage ROM. By selectively choosing and utilizing only the relevant ROMs as parents, such as excluding the ROM for the contact problem if it does not contribute significantly to the performance of the $\textrm{CO}_{2}$ storage ROM, we can optimize the training process and streamline the computational resources.

Furthermore, the third challenge also involves reaching knowledge saturation. Based on our observations, simply increasing the number of parents does not consistently lead to enhanced model performance. To illustrate, as shown in Fig. 3, having three or four parents yields roughly equivalent results. A potential solution to this issue could involve employing a pruning framework to select relevant parents.

The fourth challenge revolves around integrating physical information, either by including an additional loss function or modifying the network architecture. This entails finding ways to embed fundamental physical principles into the learning process. Given that our framework strictly relies on data, it might not yield a genuinely physical solution and could generate predictions lacking a physical basis. To overcome this issue for each individual component (each child or parent), one can employ the concept of physics-informed neural networks^47^. However, this framework may not be applicable when dealing with a combination of parents and children, each influenced by different sets of physical phenomena. In such cases, it might be beneficial to explore transfer learning techniques using physics-guided models^48^.

Lastly, it is important to highlight that our progressive learning framework follows a strict hierarchical structure. To elaborate, consider Fig. 6. If we select ROM: multiphase flow as a parent for the ROM: $\textrm{CO}_{2}$ storage, it automatically implies that ROM: transport problem becomes a grandparent. In such a scenario, if we decide to retrain ROM: transport problem, it necessitates retraining ROM: multiphase flow, followed by ROM: $\textrm{CO}_{2}$ storage. This strict hierarchical growth can introduce complexities and dependencies in the training process.

To address this challenge, one possible approach is to utilize a directed acyclic graph (DAG) that allows for information exchange among different sources without assuming a strict hierarchical connection. A DAG can provide more flexibility in incorporating information from multiple models or sources, eliminating the need for a fixed hierarchical structure. This approach has been explored in the context of machine learning models, as demonstrated in the work of Gorodetsky et al.^49^. By adopting a DAG-based approach, we can mitigate the problem of strict hierarchical dependencies and enhance the adaptability and scalability of our framework. Overall, our proposed approach will expedite research and development in subsurface physics, facilitating safer and more cost-effective decision-making for energy production, groundwater extraction, and underground storage.

## Methodology

We propose using progressive learning applied in the context of progressive ROMs (p-ROM). In our framework, we design “parent” and “child” models to tackle various challenges. Each model has unique inputs (i.e., parameters) and characteristics (i.e., physics). For example, a child model might represent the deformation of solids under certain boundary conditions. In contrast, a parent model investigates fluid dynamics, considering parameters like the Rayleigh number, diffusivity coefficients, or fluid velocity. A distinctive feature of our framework is the innovative communication method between parent and child models through hidden layers. This approach of layer-based communication enhances the efficiency of information sharing, overcoming the limitations associated with the specificity of input data and outputs. Thus, when a child model discovers functional patterns within hidden layers of parents, it can transmit these insights to the child model. This process enriches the child model’s analysis, providing a wider range of insights for its specific tasks.

This transfer of knowledge, however, is not indiscriminate. Our framework employs “information gates” that regulate this flow, ensuring that the child model is only influenced by parent model data when it proves advantageous. Should the parent’s findings be irrelevant, these gates close, blocking the transfer. This selective sharing mechanism ensures that the child model benefits from the parent’s hidden layer insights only when pertinent, potentially enhancing problem-solving abilities.

Our framework’s ability to learn adaptively from parent models and its flexible communication setup enables it to address various challenges. This versatility applies across different fields, from fluid dynamics to solid mechanics, providing a dynamic approach to problem-solving. As a result, our framework stands out as a powerful tool for enhancing problem understanding and solving efficiency.

**Figure 7:**
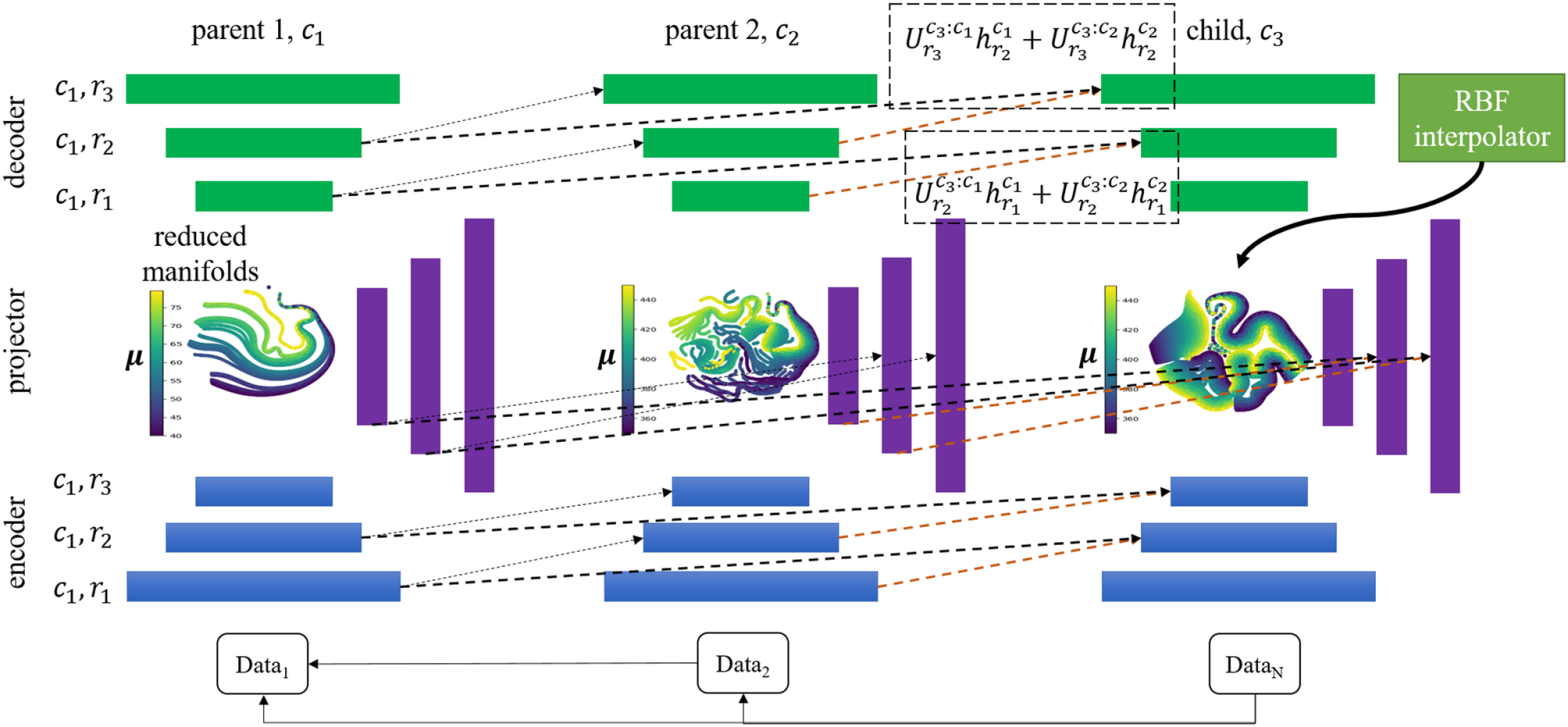
Schematic representation of the p-BT-ROM setup, comprising three main components: parent 1 ($c_1$), parent 2 ($c_2$), and the child ($c_3$). Each component is a BT-ROM model consisting of an encoder, decoder, and projector, each with three layers ($r_1$, $r_2$, $r_3$). During training/inference, the input of the child model, $\textrm{Data}_{\textrm{N}}$, is also used as input for parent 2 and parent 1. Similarly, $\textrm{Data}_{2}$ is used as input for parent 1 during the training/inference of parent 2. If parents and child have different input dimensions, a single linear layer (adapter) handles the discrepancy. The parameters of parent 1 are frozen during parent 2’s training, and the parameters of parents 1 and 2 are frozen during the child’s training. Any adapters must be trained concurrently with the child. The visualization includes t-SNE reduced manifold plots showcasing data distribution across different parameter values $\varvec{\mu }$, generated using Scikit-Learn with a perplexity setting of 15.

We apply our framework to various data-driven ROMs. In particular, we utilize the Barlow Twins reduced-order model (BT-ROM) methodology, as detailed in Supplementary sec. 3.1 and referenced in the literature^34^. Our proposed model, the progressive BT-ROM (p-BT-ROM), is outlined in Fig. 7. It features a three-column architecture, with each column representing a different model. The first two columns serve as parent models, known as parent 1 ($c_1$) and parent 2 ($c_2$), while the third column is the child model ($c_3$), which is our primary focus.

The p-BT-ROM is designed for growth, allowing adding more columns and models as needed, depending on available computational resources. This feature is crucial for handling complex datasets and challenges. Unlike traditional setups, the models within our framework don’t follow a strict order. For example, parent 2 ($c_2$) doesn’t have to follow parent 1 ($c_1$) directly. Each parent model works independently and can operate on its own. However, the child model has the unique ability to link with both parent models. These connections go beyond structure, enabling the child model to use the knowledge and insights from the parent models.

This setup creates a flexible and dynamic learning environment. By connecting with the parent models, the child model can access a wide range of knowledge and experiences. This process allows the child model to enhance problem-solving skills by incorporating what it learns from the parent models. Therefore, our framework encourages a collaborative and evolving approach to learning, overcoming the limitations of traditional models (one-to-one transfer vs. many-to-one transfer).

Our approach to progressive learning within the p-BT-ROM facilitates knowledge transfer from older models to newer ones, improving their ability to learn and perform. New models start with an advantage, using insights gained by their predecessors. This strategy helps overcome the usual hurdles of direct knowledge transfer and prevents the problem of catastrophic forgetting, where new knowledge replaces old. Instead, our models build on and refine their understanding over time, leading to more robust and more comprehensive development.

The BT-ROM architecture, as depicted in Fig. 7 comprises of an encoder, a decoder, and a projector. Each component features a series of layers or stages, denoted as $r_1, r_2, r_3, \ldots , r_n$. The function $\mathscr {N}(\cdot )$ represents any nonlinear activation function used within the model, such as tanh, Sigmoid, or ReLU. The term $\mathscr {L}(\cdot )$ refers to a linear transformation layer within each BT-ROM column, which processes the output from its preceding layer, $h_{r_i-1}^{c_k}$, applying a transformation defined by weights $\textbf{W}$ and bias $\textbf{b}$.

Additionally, ${U}(\cdot )$ is a linear transformation layer but specifically manages lateral connections between columns. It transforms the output from a preceding layer in another column, $h_{r_i-1}^{c_j}$ (where $j<k$), utilizing its own set of weights and biases. The output for a given layer $r_i$ in column $c_k$ is calculated as

1 $$\begin{aligned} h_{r_i}^{c_k}=\mathscr {N}\left( \mathscr {L}_{r_i}^{c_k} \left( h_{r_i-1}^{c_k}\right) +\sum _{j<k}\left( {U}_{r_i}^{{c_k}: {c_j}} \left( h_{r_i-1}^{c_j} \right) \right) \right) , \end{aligned}$$

For the model’s initial column ($c_k=0$), the output is given by:

2 $$\begin{aligned} h_i^{c_k=0}=\mathscr {N}\left( \mathscr {L}_i^{c_k=0} \left( h_{i-1}^{c_k=0} \right) \right) . \end{aligned}$$

This structure ensures that if the information from a previous model does not contribute to the learning process, its influence is effectively nullified by setting ${U}(\cdot )$ to zero. This mechanism allows for focused learning and reapplication of the model to previously learned tasks, thereby preventing catastrophic forgetting. For example, to make predictions based on a task learned by parent 2, the model can disregard outputs from parent 1 and the child, focusing solely on the output from parent 2. Significantly, each BT-ROM’s component—encoder, decoder, and projector—operates independently, with lateral connections present within each component but not between them.

### Supplementary Information

Supplementary Information.

## Data Availability

The authors will provide public access to these results of federally sponsored research in accordance with the DOE Public Access Plan https://www.energy.gov/downloads/doe-public-access-plan. Source code and examples will be hosted on Sandia National Laboratory Github website upon approval for information release.
