# Supplementary material for: Progressive transfer learning for advancing machine learning-based reduced-order modeling
Source: Sci Rep. 2024 Jul 8;14:15731. doi: 10.1038/s41598-024-64778-y (PMC11231231; doi:10.1038/s41598-024-64778-y)
Supplement: Supplementary file 1 — Supplementary Information. [file 41598_2024_64778_MOESM1_ESM.pdf]

Progressive transfer learning for advancing machine learning-based reduced-order modeling

Teeratorn Kadeethum[1], Daniel O'Malley[2], Youngsoo Choi[3], Hari S. Viswanathan[2], and Hongkyu Yoon[1,*]

[1]Sandia National Laboratories, Albuquerque, NM, 87185, USA
[2]Los Alamos National Laboratory, Los Alamos, NM, 87545, USA
[3]Lawrence Livermore National Laboratory, Livermore, CA, 94550, USA
[*]hyoon@sandia.gov

Supplementary Information

Supplementary information

Supplementary sec. 1 A collections of geometries used in this manuscript

Here, we summarize all meshes used throughout this manuscript in Supplementary fig. 1 and Supplementary tab. 1. In short, Supplementary fig. 1a is a 2-Dimensional domain with a size of 2×1 and has a hole inside the domain. It has 1503 nodes and is used for all physics problems (Supplementary sec. 2.1 to Supplementary sec. 2.4). Supplementary fig. 1b is also a 2-Dimensional domain with a size of 2×1 and has two holes inside the domain. It has 1698 nodes and is used for Problem #2 (Supplementary sec. 2.2). Supplementary fig. 1c is a 2-Dimensional domain with a size of 2×1 and has three holes inside the domain. It has 620 nodes and is used for Problem #3 (Supplementary sec. 2.3). Supplementary fig. 1d is a 3-Dimensional domain with a size of $1 \times 1 \times 1$ and has no hole inside the domain. It has 1330 nodes and is used for Problem #4 (Supplementary sec. 2.4). We note that the details of each physics problem can be found in Supplementary sec. 2.

Supplementary tab. 1. Summary of main information for each mesh.

	Dimension	Size	Number of nodes	Used for
A rectangular with 1 hole	2	2 x 1	1503	Problems #1, 2, 3, 4
A rectangular with 2 holes	2	2 x 1	1698	Problem #2
A rectangular with 3 holes	2	2 x 1	620	Problem #3
A cube	3	1 x 1 x 1	1330	Problem #4

Supplementary sec. 2 A collections of physical problems used in this manuscript

For all problems, let $\Omega \subset \mathbb{R}^d$ ($d \in \{1,2,3\}$) denote the computational domain and $\partial\Omega$ denote the boundary. $\partial\Omega$ can be represented by the Dirichlet boundary condition ($\partial\Omega_D$) or the Neumann boundary condition ($\partial\Omega_N$). For Problems # 1 to 3, the time domain is denoted by $\mathbb{T} = (0, \tau]$ with $\tau > 0$ (i.e., τ is the final time). We note that Problem # 4 is a steady-state problem.

Supplementary sec. 2.1 Problem #1: transport problem with velocity as a parameter

In this problem, we focus on the transport problem, and we have a concentration $c : \Omega \times \mathbb{T} \to \mathbb{R}$ (fraction) as a primary variable. Our governing equations are shown below

$$\begin{aligned} \frac{\partial}{\partial t}(\phi c) + \nabla \cdot (\boldsymbol{q} c) - \nabla \cdot (\boldsymbol{D} \nabla c) = q \quad &\text{in} \quad \Omega \times (0, \mathbb{T}], \\ \eta(\boldsymbol{q}, c) \cdot \mathbf{n} = c_{in} \boldsymbol{q} \cdot \mathbf{n} \quad &\text{on} \quad \partial\Omega_{\text{in}} \times (0, \mathbb{T}], \\ \boldsymbol{D} \nabla c \cdot \mathbf{n} = 0 \quad &\text{on} \quad \partial\Omega_{\text{out}} \times (0, \mathbb{T}], \\ c = c_0 \quad &\text{in} \quad \Omega \text{ at } t = 0, \end{aligned} \qquad \text{(Supplementary eq. 1)}$$

where ϕ is a porosity, $\boldsymbol{D}$ is a tensor of diffusivity coefficient, q represents a source term, c_{in} is the inflow concentration, c_0 is the initial concentration, $\mathbf{n}$ is a normal unit vector to each element surface, the mass flux $\eta(\boldsymbol{q}, c)$ is defined as

$$\eta(\boldsymbol{q}, c) := \boldsymbol{q} c - \boldsymbol{D}(\phi) \nabla c. \qquad \text{(Supplementary eq. 2)}$$

$\boldsymbol{q}$ is the superficial velocity vector defined by $\boldsymbol{q} = (20.0, \mu_1 \cos(\pi x))$. To approximate c_h, we use the first-order discontinuous Galerkin approximation, and the detail of discretization and the FOM source codes can be found in[1,2]. Here, we have one parameter $\boldsymbol{\mu} = (\mu_1 = [5.0, 25.0])$. The rest parameters are fixed as $\phi = 0.3$, $\boldsymbol{D} = (0.2, 0.0, 0.0, 0.2)$ (i.e., homogeneous and isotropic), and we enforce $c_0 = 0.0$ and $c_{in} = 1.0$ on the left boundary. We use geometries and meshes shown in Supplementary fig. 1.

Supplementary sec. 2.2 Problem #2: Transport problem with diffusivity coefficient as a parameter

Here, we also use the same governing equations shown in (Supplementary eq. 1). For fixed parameters, we use $\phi = 0.3$, $\boldsymbol{q} = (20.0, \cos(\pi x))$, and we enforce $c_0 = 0.0$ and $c_{in} = 1.0$ on the left boundary. The parameter $\boldsymbol{\mu} = (\boldsymbol{D} = (\mu_1, 0.0, 0.0, \mu_1))$, again, homogeneous and isotropic, and $\mu_1 = [0.1, 1.0]$. We use geometries and meshes shown in Figs. 1a, b.

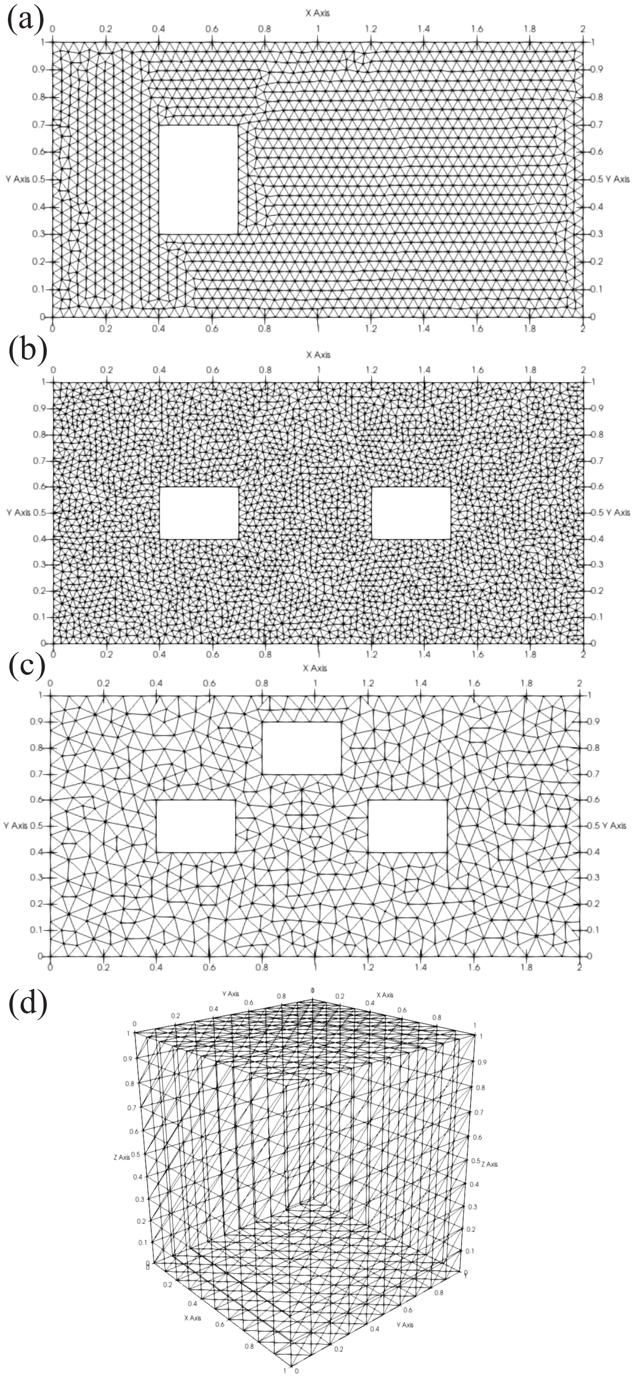

Supplementary fig. 1. Illustrations of geometries and their corresponding mesh used in this manuscript. The details of each mesh can be found in Table 1.

Supplementary sec. 2.3 Problem #3: gravity-driven problem with Rayleigh number as a parameter

For Problem #3, we use a gravity-driven flow in porous media. We follow the FOM used in[2,3], and here we briefly discuss the governing equations of this problem. Essentially, we deal with coupled PDEs of mass conservation and heat advection-diffusion equations. As presented in[2,3], we write our system of equations in dimensionless form, and primary variables in this setting are $\boldsymbol{u}(\cdot,t) : \Omega \times \mathbb{T} \to \mathbb{R}^d$, which is a vector-valued Darcy velocity (dimensionless), $p(\cdot,t) : \Omega \times \mathbb{T} \to \mathbb{R}^d$, which is a scalar-valued fluid pressure (dimensionless), and $T(\cdot,t) : \Omega \times \mathbb{T} \to \mathbb{R}^d$, which is a scalar-valued fluid temperature (dimensionless). Time is denoted as t (dimensionless). The system of our governing equations read

$$
\begin{aligned}
\boldsymbol{u}+\nabla p-\mathbf{y}\mathrm{Ra}T&=0, \quad \text{in} \quad \Omega\times\mathbb{T},\\
\nabla\cdot\boldsymbol{u}&=0, \quad \text{in} \quad \Omega\times\mathbb{T},\\
p&=p_D \quad \text{on} \quad \partial\Omega_p\times\mathbb{T},\\
\boldsymbol{u}\cdot\mathbf{n}&=q_D \quad \text{on} \quad \partial\Omega_q\times\mathbb{T},\\
p&=p_0 \quad \text{in} \quad \Omega \text{ at } t=0,
\end{aligned}
\qquad \text{(Supplementary eq. 3)}
$$

for the mass balance equation where $\partial\Omega_p$ and $\partial\Omega_q$ are the prescribed pressure and flux boundaries, respectively, and Ra is the Rayleigh number

$$
\mathrm{Ra} := \frac{g\alpha\kappa\Delta T^* H}{K}. \qquad \text{(Supplementary eq. 4)}
$$

Subsequently, the heat advection-diffusion equation is described as

$$
\begin{aligned}
\frac{\partial T}{\partial t}+\boldsymbol{u}\cdot\nabla T-\nabla^2 T-f_c&=0, \quad \text{in} \quad \Omega\times(0,\mathbb{T}],\\
T&=T_D \quad \text{on} \quad \partial\Omega_T\times(0,\mathbb{T}],\\
(-\boldsymbol{u}T+\nabla T)\cdot\mathbf{n}&=T_{\text{in}}\boldsymbol{u}\cdot\mathbf{n} \quad \text{on} \quad \partial\Omega_{\text{in}}\times(0,\mathbb{T}],\\
\nabla T\cdot\mathbf{n}&=0 \quad \text{on} \quad \partial\Omega_{\text{out}}\times(0,\mathbb{T}],\\
T&=T_0 \quad \text{in} \quad \Omega \text{ at } t=0,
\end{aligned}
\qquad \text{(Supplementary eq. 5)}
$$

where $\partial\Omega_T$ is prescribed temperature boundary, $\partial\Omega_{\text{in}}$ and $\partial\Omega_{\text{out}}$ denote inflow and outflow boundaries, respectively, defined as

$$
\partial\Omega_{\text{in}} := \{\boldsymbol{X}\in\partial\Omega : \boldsymbol{u}\cdot\mathbf{n}<0\} \quad \text{and} \quad \partial\Omega_{\text{out}} := \{\boldsymbol{X}\in\partial\Omega : \boldsymbol{u}\cdot\mathbf{n}\geq 0\}. \qquad \text{(Supplementary eq. 6)}
$$

The detail of discretization, similar to problems #1 and 2 that we use discontinuous Galerkin approximation, as well as the FOM source codes, could be found in[1,2]. We note that as our finite element solver utilizes an adaptive time-stepping[1,2], each snapshot may have a different number of time-steps N^t. We use geometries and meshes shown in Figs. 1a, c. Here, we have one parameter $\boldsymbol{\mu} = (\mu_1 = \mathrm{Ra})$, and $\mathrm{Ra} = [350.0, 450.0]$.

Supplementary sec. 2.4 Problem #4: Hyperelasticity problem with external forces as a parameter

Problem #4 focuses on the finite deformation of a hyperelastic material, which is applicable to many engineering applications, such as biomedical engineering and material science[4–6]. The weak form of mechanical equilibrium equations in the reference configuration, where the domain before deformation is represented with Ω_0 and after deformation with Ω_i, where i represents different states of deformation (i.e., $\boldsymbol{\delta}\mathbf{u} = \mathbf{u}_i - \mathbf{u}_0$), is

$$
\int_{\Omega_0} \mathbf{P} : \nabla(\boldsymbol{\delta}\mathbf{u})\, dV - \int_{\Omega_0} \mathbf{B}\cdot\boldsymbol{\delta}\mathbf{u}\, dVs - \int_{\partial\Omega_N} \mathbf{T}\cdot\boldsymbol{\delta}\mathbf{u}\, dS = 0, \qquad \text{(Supplementary eq. 7)}
$$

where $\mathbf{P}$ is the 1st Piola Kirchhoff stress tensor, which is a function of Young's modulus (E) and Poisson ratio (ν). $\mathbf{B}$ and $\mathbf{T}$ are the body and traction forces, respectively, and $\mathbf{u}$ is a displacement vector (primary variable). To approximate $\mathbf{u}$ (i.e., $\mathbf{u}_h$), following[7], we use a discontinuous Galerkin approximation of the first order. We use PETSc SNES as a nonlinear solver, and MUMPS as a linear solver[8] with absolute and relative tolerances of 1×10^{-6} and 1×10^{-16}, respectively. We utilize a backtracking line search with slope descent parameter of 1×10^{-4}, initial step length of 1.0, and quadratic order of the approximation. We utilize both 2- and 3-Dimensional domains for this problem.

Supplementary sec. 2.4.1 2-Dimensional domain

First, we use geometry and mesh shown in Figs. 1a-c. $\mathrm{E} = 100$ MPa, and $\nu = 0.46$. We set $\mathbf{B} = (0.0, 0.0)$ and $\mathbf{T} = (0.0, 0.0)$. We enforce $\mathbf{u} = (\mu_1, \mu_2)$ at the boundary condition on the face of where $y = 1.0$, top surface. Other faces have a roller boundary condition (i.e., no normal displacement). Our parameter space, here we have two parameters, $\boldsymbol{\mu} = (0.05\mu_1, 0.05\mu_2)$, and $\mu_1 = [-1.0, 1.0]$ and $\mu_2 = [-1.0, 1.0]$.

Supplementary sec. 2.4.2 3-Dimensional domain

Here, we use a geometry and mesh shown in Supplementary fig. 1d, E = 10 Pa, and $\nu = 0.3$. We set $\mathbf{B} = (0.0, -0.5, 0.0)$ and $\mathbf{T} = (\mu_1, 0.0, 0.0)$. We enforce $\mathbf{u} = (0.0, 0.0, 0.0)$ at the boundary condition on the face of where $x = 0.0$, and

$$\begin{aligned}\mathbf{u} =&(0.0,\\ &\mu_2(0.5 + (y-0.5)\cos(\pi/3) - (z-0.5)\sin(\pi/3) - y)/2,\\ &\mu_2(0.5 + (y-0.5)\sin(\pi/3) + (z-0.5)\cos(\pi/3) - x))/2)\end{aligned} \quad \text{(Supplementary eq. 8)}$$

at the boundary condition on the face of where $x = 1.0$. Other faces have a roller boundary condition (i.e., no normal displacement). Our parameter space is similar to the 2-Dimensional case as we have two parameters, $\boldsymbol{\mu} = (\mu_1, \mu_2)$, and $\mu_1 = [0.1, 0.9]$ and $\mu_2 = [0.1, 0.9]$.

Supplementary sec. 3 A collections of reduced order model used in this manuscript

Supplementary sec. 3.1 Reduced order modeling with Barlow Twins self-supervised learning

[3] has proposed a unified data-driven ROM, BT-ROM, that (1) bridges the performance gap between the linear and nonlinear manifold approaches and (2) can operate on unstructured meshes, which provides flexibility in its application to standard numerical solvers, on-site measurements, or experimental data. Even though it has been extended to boosting BT-ROM (BBT-ROM) and BT-ROM with uncertainty quantification (UQ-BT-ROM)[7,9], throughout this study, we will focus only on the BT-ROM variation.

The summary of BT-ROM is shown in Supplementary fig. 2. Our BT-ROM starts with an initialization of the training set $\boldsymbol{\mu}$, our parameter space. Subsequently, we query a FOM (see Supplementary sec. 2) for each parameter $\boldsymbol{\mu}$ in the training set. We note that the same procedures apply for validation and testing sets, but we will only discuss the training set for brevity. The third step entails a data compression stage through training BT-AE developed in[3].

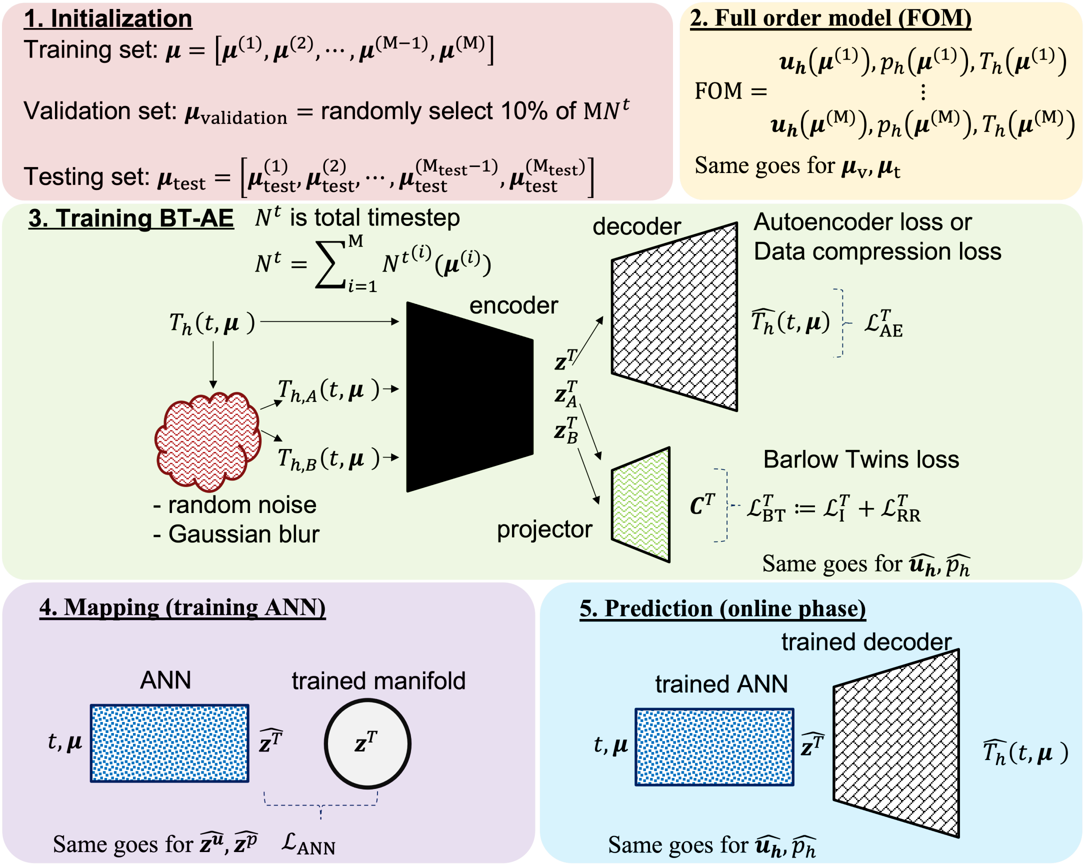

Supplementary fig. 2. The summary of procedures taken to establish the proposed BT-AE - from[3]

We illustrate our algorithm in Al. 1, but for further details and implementation. Here, we only summarize the main components; please refer to[3,7]. The training involves two tasks; the first is the training of BT (encoder and projector) - the outer loop with $\mathbf{B}_{\text{outer}}$. The second sub-task is the training of AE (encoder and decoder) - the inner loop with $\mathbf{B}_{\text{inner}}$. First, we alter our training set by creating $\boldsymbol{X}_{h,A}(t,\boldsymbol{\mu})$ and $\boldsymbol{X}_{h,B}(t,\boldsymbol{\mu})$ from $\boldsymbol{X}(t,\boldsymbol{\mu})$) through the addition of random noise

$$\widetilde{\boldsymbol{X}_{h,A}}(t,\boldsymbol{\mu}),\widetilde{\boldsymbol{X}_{h,B}}(t,\boldsymbol{\mu}) = \boldsymbol{X}(t,\boldsymbol{\mu}) + \varepsilon\,\mathrm{SD}(\boldsymbol{X}(t,\boldsymbol{\mu}))\,\mathscr{G}(0,1) \qquad \text{(Supplementary eq. 9)}$$

where $\widetilde{\boldsymbol{X}_{h,A}}(t,\boldsymbol{\mu}),\widetilde{\boldsymbol{X}_{h,B}}(t,\boldsymbol{\mu})$ are altered $\boldsymbol{X}(t,\boldsymbol{\mu})$ by (Supplementary eq. 9). The constant ε, which is set to 0.1, determines the noise level, and $\mathscr{G}(0,1)$ is a random value that is sampled from the standard normal distribution with mean and standard deviation of zero and one, respectively. Subsequently, we pass $\widetilde{\boldsymbol{X}_{h,A}}(t,\boldsymbol{\mu}),\widetilde{\boldsymbol{X}_{h,B}}(t,\boldsymbol{\mu})$ through Gaussian blur operation

$$\boldsymbol{X}_{h,A}(t,\boldsymbol{\mu}),\boldsymbol{X}_{h,B}(t,\boldsymbol{\mu}) = \frac{1}{\sqrt{2\pi\,\mathrm{SD}\left(\widetilde{\boldsymbol{X}_{h,A}}(t,\boldsymbol{\mu}),\widetilde{\boldsymbol{X}_{h,B}}(t,\boldsymbol{\mu})\right)^2}}\exp\left(-\frac{\widetilde{\boldsymbol{X}_{h,A}}(t,\boldsymbol{\mu}),\widetilde{\boldsymbol{X}_{h,B}}(t,\boldsymbol{\mu})^2}{2\,\mathrm{SD}\left(\widetilde{\boldsymbol{X}_{h,A}}(t,\boldsymbol{\mu}),\widetilde{\boldsymbol{X}_{h,B}}(t,\boldsymbol{\mu})\right)^2}\right) \qquad \text{(Supplementary eq. 10)}$$

to obtain $\boldsymbol{X}_{h,A}(t,\boldsymbol{\mu})$ and $\boldsymbol{X}_{h,B}(t,\boldsymbol{\mu})$. Next, we pass $\boldsymbol{X}_{h,A}(t,\boldsymbol{\mu})$ and $\boldsymbol{X}_{h,B}(t,\boldsymbol{\mu})$ to the encoder (it is noted we have only one encoder) resulting in $\boldsymbol{z}_A^{\boldsymbol{X}}(t,\boldsymbol{\mu})$ and $\boldsymbol{z}_B^{\boldsymbol{X}}(t,\boldsymbol{\mu})$. We then use $\boldsymbol{z}_A^{\boldsymbol{X}}(t,\boldsymbol{\mu})$ and $\boldsymbol{z}_B^{\boldsymbol{X}}(t,\boldsymbol{\mu})$ as an input to the projector resulting in the cross-correlation matrix $\mathbf{C}^{\boldsymbol{X}}(t,\boldsymbol{\mu})$. $\mathbf{C}^{\boldsymbol{X}}(t,\boldsymbol{\mu})$ is a square matrix with the dimensionality of the projector's output. The Barlow Twins loss $\mathscr{L}_{\text{BT}}^{\boldsymbol{X}}$, BT loss, is then calculated using

$$\mathscr{L}_{\text{BT}}^{\boldsymbol{X}} := \mathscr{L}_{\text{I}}^{\boldsymbol{X}} + \mathscr{L}_{\text{RR}}^{\boldsymbol{X}} \qquad \text{(Supplementary eq. 11)}$$

where

$$\mathscr{L}_{\text{I}}^{\boldsymbol{X}} := \sum_i \left(1 - \mathbf{C}_{ii}^{\boldsymbol{X}}(t,\boldsymbol{\mu})\right)^2, \qquad \text{(Supplementary eq. 12)}$$

and

$$\mathscr{L}_{\text{RR}}^{\boldsymbol{X}} := \lambda \sum_i \sum_{j\neq i} \mathbf{C}_{ij}^{\boldsymbol{X}}(t,\boldsymbol{\mu})^2. \qquad \text{(Supplementary eq. 13)}$$

Here, $\mathbf{C}_{ii}^{\boldsymbol{X}}(t,\boldsymbol{\mu})$ denotes the i-th diagonal entry of $\mathbf{C}^{\boldsymbol{X}}(t,\boldsymbol{\mu})$, λ is set to 5×10^{-3}, and $\mathbf{C}_{ij}^{\boldsymbol{X}}$ are off-diagonal entries of $\mathbf{C}^{\boldsymbol{X}}$.

Subsequently, we obtain $\boldsymbol{z}^{\boldsymbol{X}}(t,\boldsymbol{\mu})$ by passing $\boldsymbol{X}_h(t,\boldsymbol{\mu})$ to the encoder. The $\boldsymbol{z}^{\boldsymbol{X}}(t,\boldsymbol{\mu})$ is used to reconstruct $\widehat{\boldsymbol{X}}_h(t,\boldsymbol{\mu})$ through the decoder, and we calculate our data compression loss or AE loss ($\mathscr{L}_{\text{AE}}^{\boldsymbol{X}}$) using

$$\mathscr{L}_{\text{AE}}^{\boldsymbol{X}} := \mathrm{MSE}^{\boldsymbol{X}} = \frac{1}{\mathrm{M}N^t}\sum_{i=1}^{\mathrm{M}}\sum_{k=0}^{N^t}\left|\widehat{\boldsymbol{X}}_h\left(t^k,\boldsymbol{\mu}^{(i)}\right) - \boldsymbol{X}_h\left(t^k,\boldsymbol{\mu}^{(i)}\right)\right|^2. \qquad \text{(Supplementary eq. 14)}$$

We use the adaptive moment estimation (ADAM) algorithm[10] to train the framework. The learning rate (η) is calculated as[11]

$$\eta_c = \eta_{\min} + \frac{1}{2}(\eta_{\max} - \eta_{\min})\left(1 + \cos\left(\frac{\text{step}_\text{c}}{\text{step}_\text{f}}\pi\right)\right) \qquad \text{(Supplementary eq. 15)}$$

where η_c is a learning rate at step step_c, $\eta_{\min}$ is the minimum learning rate, which is set as 1×10^{-16}, $\eta_{\max}$ is the initial learning rate, which is selected as 1×10^{-5}, step_c is the current step, and step_f is the final step. To prevent our networks from overfitting behavior, we follow early stopping and generalized cross-validation criteria[12,13]. Note that instead of literally stopping our training cycle, we only save the set of trained weights and biases from being used in the online phase when the current validation loss is lower than the lowest validation from all the previous training cycles.

Algorithm 1 Training autoencoder (AE) with Barlow Twins (BT) self-supervised learning (BT-ROM) - from[7]

\# Training data $\boldsymbol{X}_h$ and distorted data $\boldsymbol{X}_{h,A}$, $\boldsymbol{X}_{h,B}$ are input of encoder
\# latent spaces $\boldsymbol{z^X}$, $\boldsymbol{z}_A^{\boldsymbol{X}}$, and $\boldsymbol{z}_B^{\boldsymbol{X}}$ are output encoder
\# latent space $\boldsymbol{z^X}$ is output of decoder
\# Approximation of $\boldsymbol{X}_h$, i.e., $\widehat{\boldsymbol{X}_h}$ is output of decoder
\# latent spaces $\boldsymbol{z}_A^{\boldsymbol{X}}$ and $\boldsymbol{z}_B^{\boldsymbol{X}}$ are input of projector
\# cross-correlation matrix $\mathbf{C}^{\boldsymbol{X}}$ is output of projector

1: Initialize (or load pre-trained models) encoder, decoder, and projector ▷ size of latent space Q has to be specified.
2: Initialize (or load pre-trained optimizers) three optimizers for each of encoder, decoder, and projector
3: Load training set $\boldsymbol{\mu}$ ▷ the total training data is $\mathrm{M}N^t$
4: Randomly select 5% of $\mathrm{M}N^t$ for validation set $\boldsymbol{\mu}_{\text{validation}}$ ▷ the total training data becomes 95% of $\mathrm{M}N^t$
5: Add random noise ▷ see (Supplementary eq. 9)
6: Add Gaussian blur ▷ see (Supplementary eq. 10)
7: From step 5 and 6, we obtain $\boldsymbol{X}_{h,A}(t,\boldsymbol{\mu})$ and $\boldsymbol{X}_{h,B}(t,\boldsymbol{\mu})$ from $\boldsymbol{X}(t,\boldsymbol{\mu})$
8: **for each** epoch **do**
9:     Outer loop: training BT ▷ Batch size $\mathbf{B}_{\text{outer}}$
10:     **for each** $\mathbf{B}_{\text{outer}}$ **do**
11:         $\boldsymbol{z}_A^{\boldsymbol{X}}(t,\boldsymbol{\mu}) = \text{encoder}\left(\boldsymbol{X}_{h,A}(t,\boldsymbol{\mu})\right)$
12:         $\boldsymbol{z}_B^{\boldsymbol{X}}(t,\boldsymbol{\mu}) = \text{encoder}\left(\boldsymbol{X}_{h,B}(t,\boldsymbol{\mu})\right)$
13:         $\mathbf{C}^{\boldsymbol{X}}(t,\boldsymbol{\mu}) = \text{projector}\left(\boldsymbol{z}_A^{\boldsymbol{X}}(t,\boldsymbol{\mu}), \boldsymbol{z}_B^{\boldsymbol{X}}(t,\boldsymbol{\mu})\right)$
14:         Calculate BT loss $\mathscr{L}_{\text{BT}}^{\boldsymbol{X}}$ ▷ see (Supplementary eq. 11)
15:         Back-propagation of BT loss w.r.t. each encoder $(\mathbf{W},\mathbf{b})$ and projector $(\mathbf{W},\mathbf{b})$
16:         Update encoder $(\mathbf{W},\mathbf{b})$ and projector $(\mathbf{W},\mathbf{b})$ using BT optimizer
17:         Update learning rate η_c of BT optimizer ▷ see (Supplementary eq. 15)
18:         Inner loop: training AE ▷ Batch size $\mathbf{B}_{\text{inner}}$
19:         **for each** $\mathbf{B}_{\text{inner}}$ **do**
20:             $\boldsymbol{z^X}(t,\boldsymbol{\mu}) = \text{encoder}\left(\boldsymbol{X}_h(t,\boldsymbol{\mu})\right)$
21:             $\widehat{\boldsymbol{X}_h}(t,\boldsymbol{\mu}) = \text{decoder}\left(\boldsymbol{z^X}(t,\boldsymbol{\mu})\right)$
22:             Calculate AE loss $\mathscr{L}_{\text{AE}}^{\boldsymbol{X}}$ (data compression loss) ▷ see (Supplementary eq. 14)
23:             Back-propagation of AE loss w.r.t. each encoder $(\mathbf{W},\mathbf{b})$ and decoder $(\mathbf{W},\mathbf{b})$
24:             Update encoder $(\mathbf{W},\mathbf{b})$ and decoder $(\mathbf{W},\mathbf{b})$ using AE optimizer
25:             Update learning rate η_c of AE optimizer ▷ see (Supplementary eq. 15)
26:         **end for**
27:     **end for**
28: **end for**

Reflecting the third step in Supplementary fig. 2.

We reiterate the autoencoder's architecture used for BT-ROM in Supplementary tab. 2; please refer to[3,7] for further details on architecture search and hyper-parameters tuning. To sum up, the BT-ROM model is composed of one encoder, one decoder, and one projector with their own sets of weight matrices (**W**) and biases (**b**). Each linear layer in Supplementary tab. 2 is subjected to the LeakyReLU activation function with a negative slope of 0.2. No batch normalization or dropout layers are used in this architecture, making it preferable and lightweight.

For the next step, we map t and $\boldsymbol{\mu}$ to its representation in the linear and nonlinear manifold $\boldsymbol{z^X}(t,\boldsymbol{\mu})$. Note that if we deal with steady-state problems, we simply drop the t term. We follow a procedure proposed by[3] and use linear radial basis function (RBF) interpolation[14] to map t and $\boldsymbol{\mu}$ to $\boldsymbol{z^X}(t,\boldsymbol{\mu})$. During the online phase, we utilize the trained RBF and the trained decoder to approximate $\widehat{\boldsymbol{X}}_h(\cdot;t,\boldsymbol{\mu})$ for each inquiry (i.e., a value of $\boldsymbol{\mu}$) through

$$\widehat{\boldsymbol{z}}^{\boldsymbol{X}}(t,\boldsymbol{\mu}) = \text{RBF}(t,\boldsymbol{\mu}), \qquad \text{(Supplementary eq. 16)}$$

and, subsequently,

$$\widehat{\boldsymbol{X}}_h(t,\boldsymbol{\mu}) = \text{decoder}\left(\widehat{\boldsymbol{z}}^{\boldsymbol{X}}(t,\boldsymbol{\mu})\right). \qquad \text{(Supplementary eq. 17)}$$

Supplementary tab. 2. Autoencoder - reflecting the third step in Supplementary fig. 2. (input and output sizes are represented by [B, DOF]. B is a batch size, and z is nonlinear manifolds.) - from[7]

**block**	**input size**	**output size**
1st linear layer	[B, DOF]	[B, int(DOF/2)]
2nd linear layer	[B, int(DOF/2)]	[B, int(DOF/4)]
3rd linear layer	[B, int(DOF/4)]	[B, int(DOF/8)]
4th linear layer	[B, int(DOF/8)]	[B, int(DOF/16)]
5th linear layer	[B, int(DOF/16)]	[B, int(DOF/32)]
1st **bottleneck**	reshape([B, int(DOF/32)])	[B, z]
2nd **bottleneck**	[B, z]	reshape([B, int(DOF/32)])
6th linear layer	[B, int(DOF/32)]	[B, int(DOF/16)]
7th linear layer	[B, int(DOF/16)]	[B, int(DOF/8)]
8th linear layer	[B, int(DOF/8)]	[B, int(DOF/4)]
9th linear layer	[B, int(DOF/4)]	[B, int(DOF/2)]
10th linear layer	[B, int(DOF/2)]	[B, DOF]

Supplementary sec. 4 Data generation

Here, we briefly explain how we create our training, validation, and testing sets. We divide our investigation into two phases. In the first phase, we focus on different physics but similar topology (Sec. Similar topology in the main text). Here, we illustrate the effects of the number of training samples and the number of parents on the BT-ROM's and p-BT-ROM's performance. In the second phase, we use different physics and topologies (Sec. Different topologies in the main text). For this phase, we only focus on the effect of the number of parents on the BT-ROM's and p-BT-ROM's performance. We have described each topology in detail in Supplementary sec. 1.

In terms of physics problems utilized throughout this study, we have four main problems. We have described each problem in detail in Supplementary sec. 2. The first one (Problem #1) is a transport problem with given different velocity fields, i.e., $\boldsymbol{q}$ is the superficial velocity vector defined by $\boldsymbol{q} = (20.0, \mu_1 \cos(\pi x))$, and our parameter $\boldsymbol{\mu} = (\mu_1 = [5.0, 25.0])$ Supplementary sec. 2.1). For this problem, we use a mesh provided in Supplementary fig. 1a, and a sample of this problem is shown in Supplementary fig. 3a. The second problem (Problem #2) is a transport problem with given different diffusivity coefficients, i.e., $\boldsymbol{\mu} = (\boldsymbol{D} = (\mu_1, 0.0, 0.0, \mu_1))$ and $\mu_1 = [0.1, 1.0]$ (Supplementary sec. 2.2). For this problem, we use two meshes presented in Figs. 1a, b, and their samples are illustrated in Supplementary fig. 3b and Supplementary fig. 4a, respectively.

The third problem (Problem #3) is a gravity-driven flow in porous media with the Rayleigh number (Ra) as a parameter $\boldsymbol{\mu} = (\mu_1 = \mathrm{Ra})$, and $\mathrm{Ra} = [350.0, 450.0]$ (Supplementary sec. 2.3). For this problem, we use two meshes presented in Figs. 1a, c, and their samples are illustrated in Supplementary fig. 3c and Supplementary fig. 4b, respectively. The fourth problem (Problem #4) is the finite deformation of a hyperelastic material (Supplementary sec. 2.4). For this problem, $\boldsymbol{\mu}$ is different for the problem in a 2-Dimensional domain, mesh: Supplementary fig. 1a and sample: Supplementary fig. 3d, and a 3-Dimensional domain, mesh: Supplementary fig. 1d and sample: Supplementary fig. 4c. For the 2-Dimensional setting, we enforce $\mathbf{u} = (\mu_1, \mu_2)$ at the boundary condition on the face of where $y = 1.0$, top surface, and $\boldsymbol{\mu} = (0.05\mu_1, 0.05\mu_2)$, and $\mu_1 = [-1.0, 1.0]$ and $\mu_2 = [-1.0, 1.0]$. For the 3-Dimensional setting, we have traction forces $\mathbf{T} = (\mu_1, 0.0, 0.0)$, and we enforce

$$\begin{aligned}\mathbf{u} =&(0.0,\\ &\mu_2(0.5 + (y-0.5)\cos(\pi/3) - (z-0.5)\sin(\pi/3) - y)/2,\\ &\mu_2(0.5 + (y-0.5)\sin(\pi/3) + (z-0.5)\cos(\pi/3) - x))/2)\end{aligned} \quad \text{(Supplementary eq. 18)}$$

at the boundary condition on the face of where $x = 1.0$. $\boldsymbol{\mu} = (\mu_1, \mu_2)$, and $\mu_1 = [0.1, 0.9]$ and $\mu_2 = [0.1, 0.9]$.

We generate training, validation, and test sets to train and evaluate our model. For the training set, we uniformly sample our parameter space $\boldsymbol{\mu}$ with M snapshots (i.e., assuming $\boldsymbol{\mu} = [\mu_1]$, $\mu_1 = (0.0, 1.0)$, and M = 3, our $\mu_1 = [0.0, 0.5, 1.0]$). For Problems #1 to #3, our actual training set would be $\mathrm{M}N_t$ (i.e., M snapshots × number of time-steps for each M). We design our framework this way to handle data provided from FOM with adaptive time-stepping, which is essential for an advection-dominated problem (i.e., to satisfy Courant–Friedrichs–Lewy (CFL) condition, Problems #1 to #3). The framework can also deliver the solution at any time, including times that do not exist in the training set. To elaborate, this does not imply that our model can extrapolate, but it can deliver any timestamps inside the range of the training set (see[2,3] for more details on this). For Problem #4, the actual training set is M because it is a steady-state problem. We then randomly select 5% of the

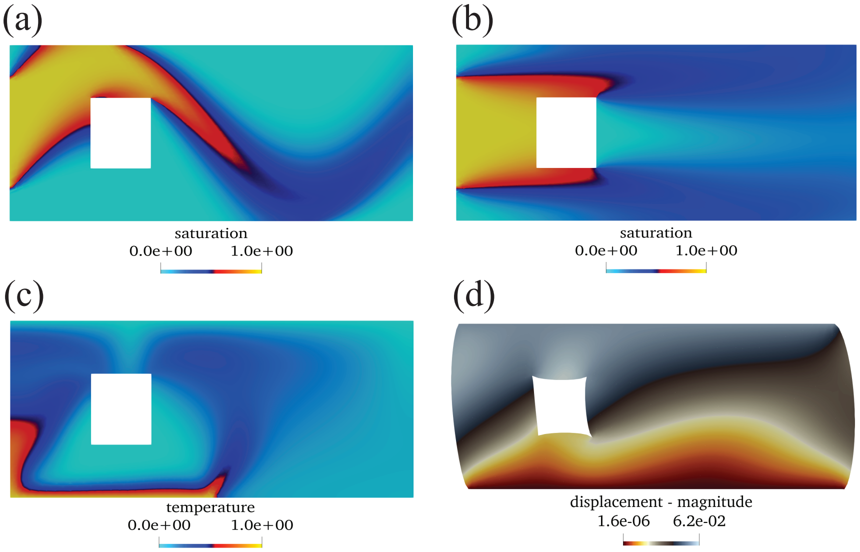

Supplementary fig. 3. Samples of physics problems simulated using mesh provided in Supplementary fig. 1a (similar topology): (a) Problem #1 (Supplementary sec. 2.1), (b) Problem #2 (Supplementary sec. 2.2), (c) Problem #3 (Supplementary sec. 2.3), and (d) Problem #4 (Supplementary sec. 2.4). We note that problems #1 to #3 are time-dependent problems while Problem #4 is at a steady-state. For (a) - (c), we show the last time-step.

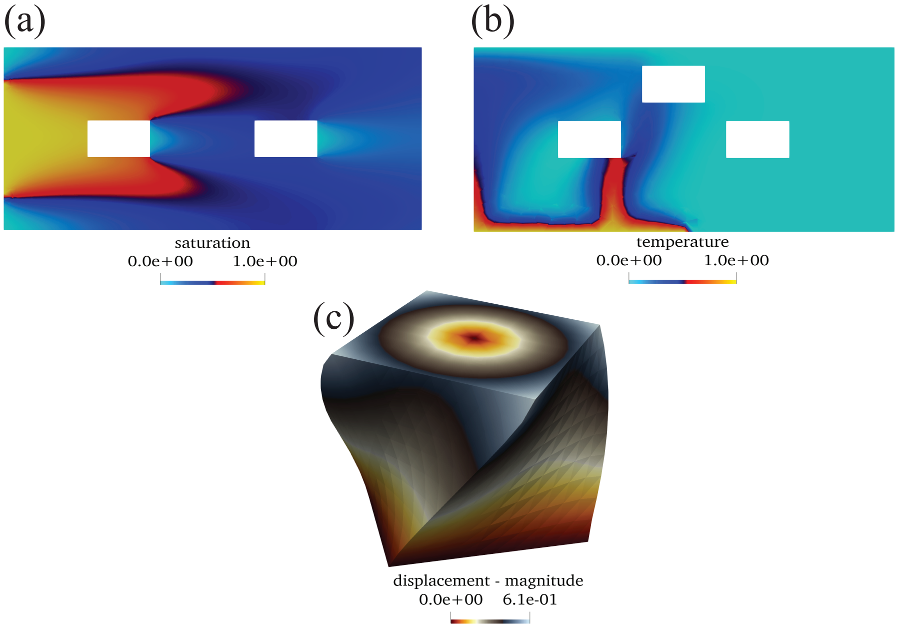

Supplementary fig. 4. Samples of physics problems simulated using different meshes provided in Supplementary fig. 1b-d (different topologies): (a) Problem #2 (Supplementary sec. 2.2) with mesh shown in Supplementary fig. 1b, (b) Problem #3 (Supplementary sec. 2.3) with mesh shown in Supplementary fig. 1c, and (c) Problem #3 (Supplementary sec. 2.4) with mesh shown in Supplementary fig. 1d. We note that problems #2 and #3 are time-dependent problems while Problem #4 is at a steady-state. For (a) - (b), we show the last time-step.

training samples to be used as a validation set. For Problems #1 to #3, the final total training samples are 0.95MN^t while, for Problem #4, the final total training samples are 0.95M For the testing set, we randomly sample our parameter space $\boldsymbol{\mu}$ with $\mathrm{M_{test}}$ snapshots.

Supplementary sec. 5 Complementary information on numerical experiments

We present supplement information on numerical experiments throughout the following sections. These results are used to support numerical examples in the main text, Sec. Results. Throughout this section, we use The mean squared error (MSE)

$$\mathrm{MSE} = \frac{1}{\mathrm{M}N^t}\sum_{i=1}^{\mathrm{M}}\sum_{j=0}^{N^t}\left|\widehat{\boldsymbol{X}}_h\left(t^j,\boldsymbol{\mu}^{(i)}\right)-\boldsymbol{X}_h\left(t^j,\boldsymbol{\mu}^{(i)}\right)\right|^2, \quad \text{(Supplementary eq. 19)}$$

and mean absolute error (MAE)

$$\mathrm{MAE} = \frac{1}{\mathrm{M}N^t}\sum_{i=1}^{\mathrm{M}}\sum_{j=0}^{N^t}|\widehat{\boldsymbol{X}}_h\left(t^j,\boldsymbol{\mu}^{(i)}\right)-\boldsymbol{X}_h\left(t^j,\boldsymbol{\mu}^{(i)}\right)|. \quad \text{(Supplementary eq. 20)}$$

as our evaluation matrices.

Supplementary sec. 5.1 Using trained models for an initialization

First, we use trained models' weights and biases to initialize a new model. If we use more than one trained model, we simply take an arithmetic average of each layer for the new model. For illustration purposes, throughout this section, we use only one trained model - Problem #1 (Supplementary sec. 2.1) - to initialize the new model. Our results are presented in Supplementary fig. 5 for the reconstruction loss of the validation set ((Supplementary eq. 14)) and Supplementary fig. 6 for the Barlow Twins loss ((Supplementary eq. 11)) of the validation set. To emphasize this process, we pick the weights and biases of the trained model that delivers the least validation loss, shown in a red circle in Figs. 5a and 6a, for the initialization. In short, from these figures, one can observe that using the trained model as a starting point, the BT-ROM delivers lower reconstruction validations loss for Problem #2 to #4 (Supplementary sec. 2.1 to Supplementary sec. 2.4). However, this trend is not always true for the Barlow Twins loss, as using the trained model as a starting point can cause an adverse effect, especially for Problems #3 and #4.

Supplementary sec. 5.2 Progressive Barlow Twins reduced order modeling without initialization

Next, we investigate the effect of using p-BT-ROM (see Sec. Methodology in the main text and Supplementary sec. 3.1) on the validation losses: the reconstruction loss of the validation set ((Supplementary eq. 14)) and the Barlow Twins loss ((Supplementary eq. 11)) of the validation set. We emphasize that here, we do not use the trained models' (or parents') weights and biases to initialize our p-BT-ROM. The results of reconstruction (Supplementary fig. 7) and Barlow Twins (Supplementary fig. 8)losses are in line; so, we will only discuss the reconstruction loss results. The schematic of p-BT-ROM specifies child∼parent(s) relationship is shown in Supplementary fig. 7a. To elaborate, for Supplementary fig. 7b, we test our model using Problem #2 (Supplementary sec. 2.2), and our p-BT-ROM has only one parent, Problem #1. One can see that p-BT-ROM delivers more accurate results.

For Supplementary fig. 7c, we test our model using Problem #3 (Supplementary sec. 2.3), and the p-BT-ROM has two parents, Problem #1 and Problem #2. Again, the validation loss of p-BT-ROM is much lower than that of BT-ROM (no parent). Lastly, we use Problem #4 (Supplementary sec. 2.4) to test our model, and the results align with previous observations. Hence, from these experiments, we observe that using p-BT-ROM can substantially improve the model accuracy.

Supplementary sec. 5.3 Progressive Barlow Twins reduced order modeling with initialization

As previously illustrated that using a trained model as an initialization (Supplementary sec. 5.1) as well as a progressive reduced order model (Supplementary sec. 5.2) generally increases our BT-ROM accuracy. Throughout this section, we investigate the p-BT-ROM with initialization (p-BT-ROM with init.) effects on the model's accuracy (i.e., investigate the effect of using p-BT-ROM (see Sec. Methodology in the main text and Supplementary sec. 3.1) on the validation losses: the reconstruction loss ((Supplementary eq. 14)) of the validation set and the Barlow Twins loss ((Supplementary eq. 11)) of the validation set). To reiterate, if we use more than one trained model (i.e., p-BT-ROM has more than one parent model), we take an arithmetic average of each layer for all parents for the new model.

We present the reconstruction loss ((Supplementary eq. 14)) of the validation set and the Barlow Twins loss ((Supplementary eq. 11)) of the validation set in Figs. 9 and 10, respectively. The schematic of p-BT-ROM specifies child∼parent(s) relationship is shown in Supplementary fig. 9a. For Figs. 9b and 10b, we focus on Problem #2 (Supplementary sec. 2.2) by using Problem #1 (Supplementary sec. 2.1) as a parent. We observe that for the reconstruction loss using p-BT-ROM and p-BT-ROM with init. are quite similar; however, p-BT-ROM with init. delivers the best Barlow Twins loss result.

For Supplementary fig. 9c and 10c, we focus on Problem #3 (Supplementary sec. 2.3) with Problem #1 and #2 as its parents. Here, the reconstruction and Barlow Twins loss using p-BT-ROM and p-BT-ROM with init. are not much different and are best.

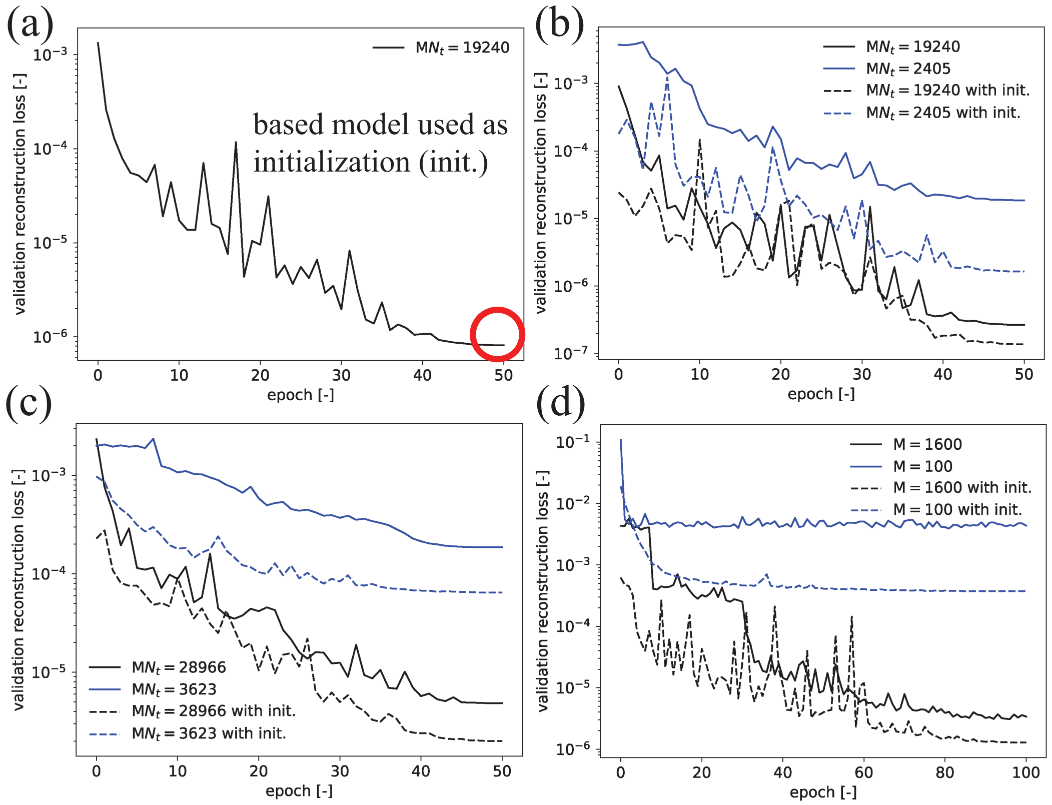

Supplementary fig. 5. Using a trained model for an initialization: validation reconstruction loss ((Supplementary eq. 14)) results of (a) physical Problem #1 (Supplementary sec. 2.1) used as an initialization for the following physical problems, (b) physical Problem #2 (Supplementary sec. 2.2) for both large dataset ($MN_t = 19240$) and small dataset ($MN_t = 2405$), (c) physical Problem #3 (Supplementary sec. 2.3) for both large dataset ($MN_t = 28966$) and small dataset ($MN_t = 3623$), and (d) physical Problem #4 (Supplementary sec. 2.4) for both large dataset ($M = 1600$) and small dataset ($M = 100$). We note that problems #1 to #3 are time-dependent problems while Problem #4 is at a steady-state. The red circle in (a) represents the epoch that has the lowest validation loss; and subsequently, be used as an initialization for the other problems.

Lastly, For Supplementary fig. 9d and 10d, we focus on Problem #4 (Supplementary sec. 2.4) with Problem #1, #2, and #3 as its parents. For this problem, the p-BT-ROM with init. delivers the best result for the reconstruction loss, while the p-BT-ROM delivers the best result for the Barlow Twins loss.

Supplementary sec. 5.4 Discussion on supplement information on numerical experiments

From Supplementary sec. 5.1, Supplementary sec. 5.2, and Supplementary sec. 5.3, we can see that by using a trained model as an initialization (init.), p-BT-ROM, and p-BT-ROM with init. can improve our BT-ROM's accuracy. However, p-BT-ROM and p-BT-ROM with init. seem to provide a better result compared to using init. alone. However, there is no clear winner between these two approaches. Hence, for all numerical examples shown in Sec. Results in the main text, we use p-BT-ROM with init. We note that the computation cost of init. is negligible compared to the training p-BT-ROM itself.

Supplementary sec. 5.5 Supplement information of similar topology section (Sec. Similar topology in the main text)

Here, we investigate p-BT-ROM's performance using one mesh, see Supplementary fig. 1a, for physics problems, Problems #1 to #4 (test model performance on different physics but similar mesh or topology).

Supplementary sec. 5.5.1 Gravity-driven in porous media - Problem #3

Throughout this section, we focus on a gravity-driven in porous media problem - Problem #3 (Supplementary sec. 2.3). One sample of this problem is shown in Supplementary fig. 3c. We aim to investigate the model's performance on a test set ($M_{test}N_t = 7260$, $M_{test} = 10$) with different training set sizes and a number of parents. This problem has three primary variables, fluid pressure, fluid velocity, and fluid temperature. Since our BT-ROM is non-intrusive, we only pick the fluid temperature

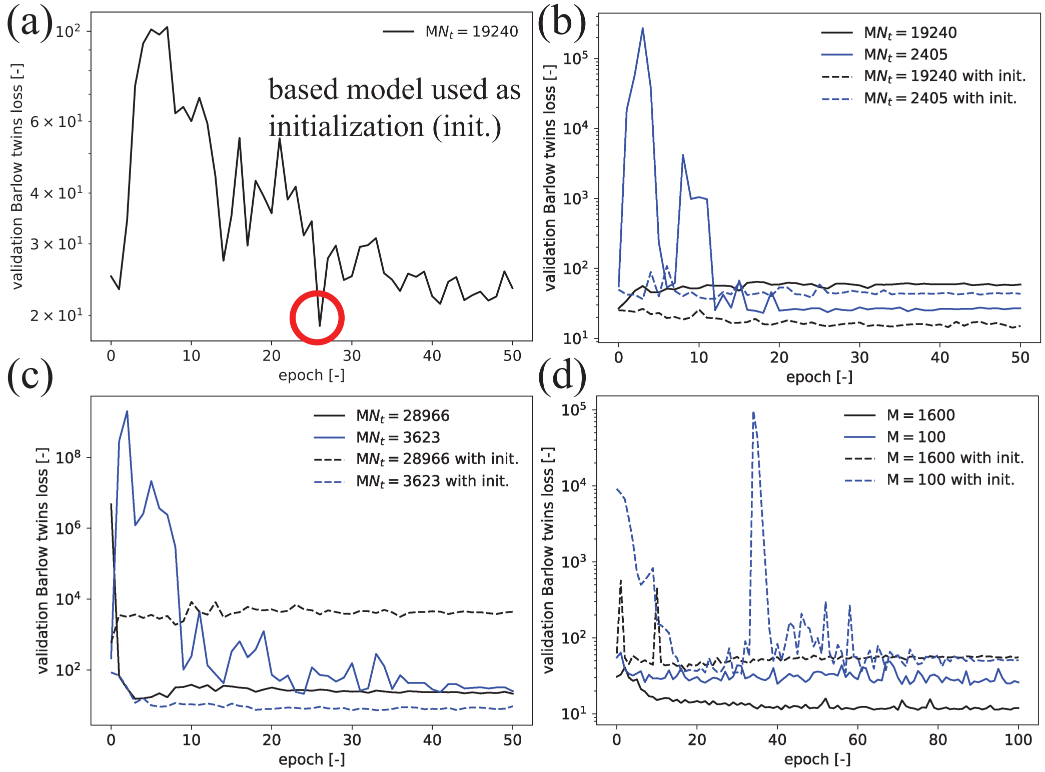

Supplementary fig. 6. Using a trained model for an initialization: validation Barlow Twins loss ((Supplementary eq. 11)) results of (a) physical Problem #1 (Supplementary sec. 2.1) used as an initialization for the following physical problems, (b) physical Problem #2 (Supplementary sec. 2.2) for both large dataset ($MN_t = 19240$) and small dataset ($MN_t = 2405$), (c) physical Problem #3 (Supplementary sec. 2.3) for both large dataset ($MN_t = 28966$) and small dataset ($MN_t = 3623$), and (d) physical Problem #4 - 2-Dimensional domain (Supplementary sec. 2.4) for both large dataset ($M = 1600$) and small dataset ($M = 100$). We note that problems #1 to #3 are time-dependent problems while Problem #4 is at a steady-state. The red circle in (a) represents the epoch that has the lowest validation loss; and subsequently, be used as an initialization for the other problems.

as our quantity of interest. The MSE ((Supplementary eq. 19)) and MAE ((Supplementary eq. 20)) results are presented in Supplementary fig. 11. The schematic of p-BT-ROM specifies child∼parent(s) relationship is shown in Supplementary fig. 12a. For the model with 1 parent, we use Problem #1 (Supplementary sec. 2.1) as a parent with a training set of $MN_t = 2405$ ($M = 5$, i.e., small dataset in Supplementary sec. 5). For the model with 2 parents, Problems #1 and #2 (Supplementary sec. 2.2) with both have a training set of $MN_t = 2405$ ($M = 5$, i.e., small dataset in Supplementary sec. 5).

From Supplementary fig. 11, we observe that the model with no parent and has a small training set ($MN_t = 3623$, $M = 5$), shown in red, performs the worst. As we add more parents, shown in blue and green, the models gain their capability and deliver better accuracy. However, even the model with two parents, shown in blue, still could not achieve the same level of accuracy as the model that trained with a large training set ($MN_t = 28966$, $M = 40$), but has zero parent.

Figs. 12b-c illustrate MAE as as a function of parameter $\boldsymbol{\mu}$ for three different cases; large dataset ($MN_t = 28966$, $M = 40$) with 0 parent, small dataset ($MN_t = 3623$, $M = 5$) with 0 parent, and small dataset ($MN_t = 3623$, $M = 5$) with 2 parents, respectively. One can observe that the pattern of these three figures is similar (i.e., the shape), but the magnitude of the MAE is different. This observation implies that the p-BT-ROM only alters the magnitude of the error, not its pattern.

Supplementary sec. 5.5.2 Hyperelasticity problem - Problem #4

Next, we move to a physics problem of finite deformation of hyperelastic material - Problem #4 (Supplementary sec. 2.4). One sample of this problem is shown in Supplementary fig. 3d. Here, we want to investigate the model's performance by using a solid mechanics at a steady-state solution (i.e., all parents, Problems #1 to #3, are transient fluid mechanics problems). We use a test set of $M_{\text{test}} = 100$. with different training set sizes and a number of parents. The quantity of interest, here, is a magnitude of displacement.

The MSE and MAE are shown in Supplementary fig. 13, and the schematic of p-BT-ROM specifies child∼parent(s) relationship is shown in Supplementary fig. 14a. It is similar to the previous section. For the model with 1 parent, we use

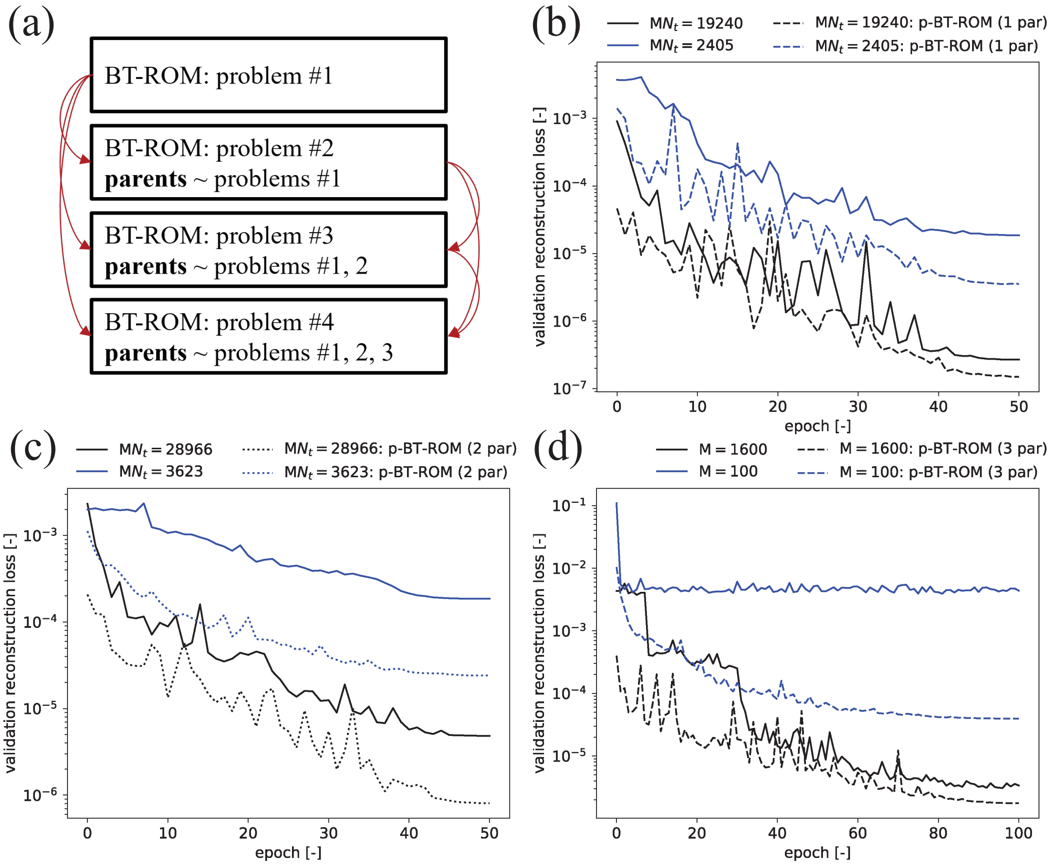

Supplementary fig. 7. p-BT-ROM without initialization: (a) schematic of p-BT-ROM specifies child∼parent(s) relationship, validation reconstruction loss ((Supplementary eq. 14)) results of (b) physical Problem #2 (Supplementary sec. 2.2) for both large dataset ($MN_t = 19240$) and small dataset ($MN_t = 2405$), (c) physical Problem #3 (Supplementary sec. 2.3) for both large dataset ($MN_t = 28966$) and small dataset ($MN_t = 3623$), and (d) physical Problem #4 - 2-Dimensional domain (Supplementary sec. 2.4) for both large dataset ($M = 1600$) and small dataset ($M = 100$). We note that problems #1 to #3 are time-dependent problems while Problem #4 is at a steady-state.

Problem #1 (Supplementary sec. 2.1) as a parent with a training set of $MN_t = 2405$ ($M = 5$), for the model with 2 parents, Problems #1 and #2 (Supplementary sec. 2.2) with both have a training set of $MN_t = 2405$ ($M = 5$), for the model with 3 parents, we add Problem #3 (Supplementary sec. 2.3) with a training set of $MN_t = 3623$ ($M = 5$). We note that all parents are trained using a small dataset (more details can be found in Supplementary sec. 5).

From Supplementary fig. 13, again, we observe that the model with no parent and has a small training set ($M = 100$), shown in red, performs the worst, while the model with a large training set ($M = 1600$) delivers the best accuracy. However, with a small training set, as we add more parents, shown in blue, green, and purple, the models gain their capability and deliver better accuracy. However, we speculate that it would be challenging to reach the same level of accuracy as that of the large dataset, even if we add even more parents.

Similar to the previous section as we use Figs. 14b-c to present the MAE results as a function of parameter $\boldsymbol{\mu}$ for three different cases; large dataset ($M = 1600$) with 0 parent, small dataset ($M = 100$) with 0 parent, and small dataset ($M = 100$) with 3 parents, respectively. Again, one can observe that the pattern of these three figures is similar (i.e., the shape), but the magnitude of the MAE is different. This observation implies that the p-BT-ROM only alters the magnitude of the error, not its pattern.

Supplementary sec. 6 Summary of number of parameters of each model

We present the number of parameters of the (p-)BT-ROM with different numbers of parents in Supplementary tab. 3 for Supplementary fig. 2 and Supplementary tab. 4 for Supplementary fig. 3. We observe that, as expected, as we increase the number of parents, the more parameters the models contain (i.e., more gates required to control the flow of information). We

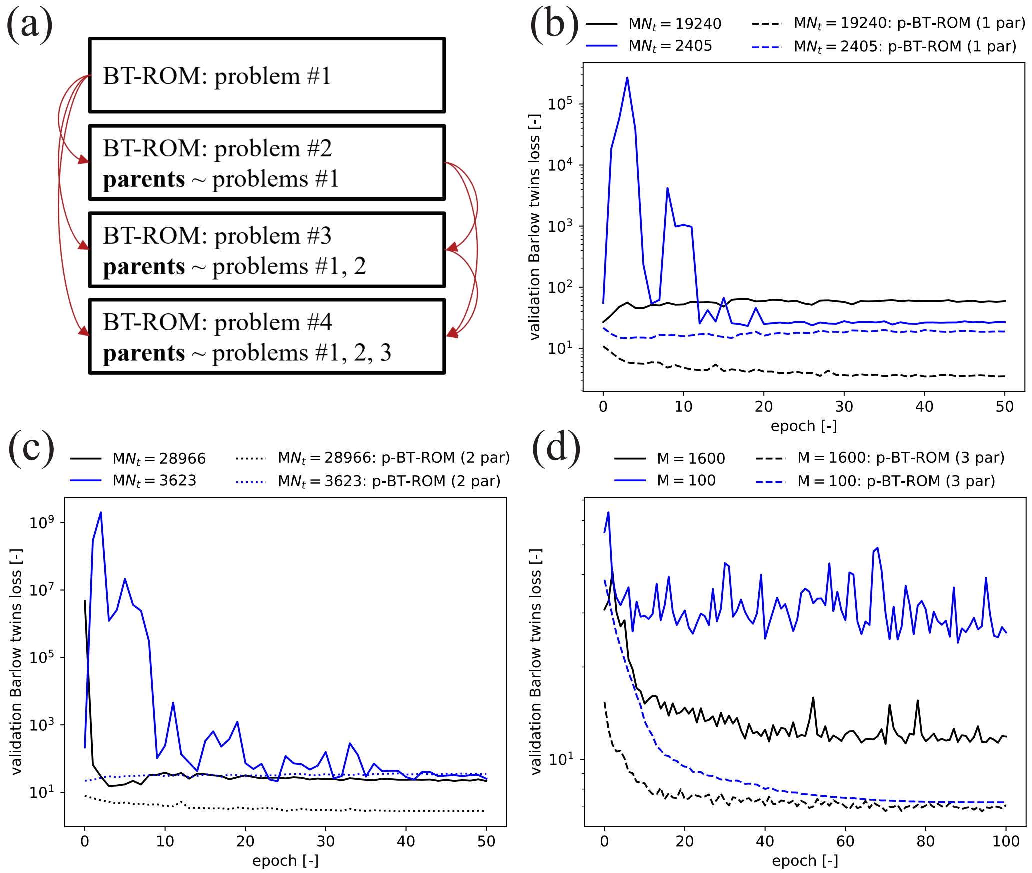

Supplementary fig. 8. p-BT-ROM without initialization: (a) schematic of p-BT-ROM specifies child∼parent(s) relationship, validation Barlow Twins loss ((Supplementary eq. 11)) results of (b) physical Problem #2 (Supplementary sec. 2.2) for both large dataset ($MN_t = 19240$) and small dataset ($MN_t = 2405$), (c) physical Problem #3 (Supplementary sec. 2.3) for both large dataset ($MN_t = 28966$) and small dataset ($MN_t = 3623$), and (d) physical Problem #4 - 2-Dimensional domain (Supplementary sec. 2.4) for both large dataset ($M = 1600$) and small dataset ($M = 100$). We note that problems #1 to #3 are time-dependent problems while Problem #4 is at a steady-state.

also note that even though the number of parameters increases. The training and prediction costs are not much different. We train and test our model using NVIDIA Quadro RTX 8000.

Supplementary tab. 3. Summary of the number of parameters of each model presented in Fig. 2 in the main text.

parents	encoder	decoder	projector
0	47,744,112	47,752,562	11,168
1	59,647,413	95,500,636	21,728
2	71,550,714	143,248,710	32,288
3	83,454,015	190,996,784	42,848

Supplementary sec. 7 Complementary information on numerical results for different topologies section (Sec. Different topologies in the main text)

Supplementary fig. 15 is used to showing the pattern of MAE as a function of parameter $\boldsymbol{\mu}$. We discuss this figure in Sec. Different topologies in the main text.

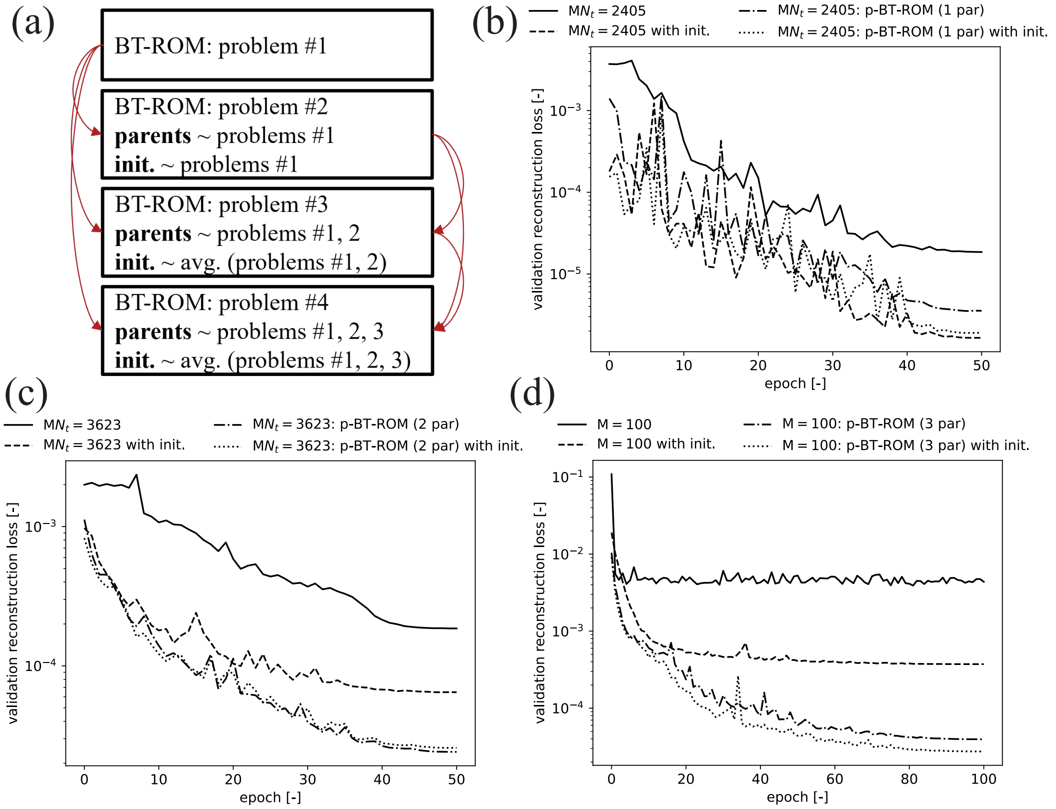

Supplementary fig. 9. Overview of p-BT-ROM Implementation and Evaluation: (a) Illustrates the p-BT-ROM framework, detailing the child-to-parent(s) relationships and the initialization process. (b) Presents the validation reconstruction loss, as defined in (Supplementary eq. 14), for Physical Problem #2 (Supplementary sec. 2.2), comparing the performance of BT-ROM, BT-ROM with initialization, p-BT-ROM, and p-BT-ROM with initialization on a small dataset ($MN_t = 2405$). (c) Shows similar validation loss results for Physical Problem #3 (Supplementary sec. 2.3), with a dataset size of $MN_t = 3623$. (d) Demonstrates the validation loss for Physical Problem #4 - A 2-Dimensional domain (Supplementary sec. 2.4) across the same ROM configurations for a dataset with $M = 100$. It is noted that Physical Problems #1 to #3 are time-dependent, whereas Problem #4 is evaluated in a steady-state condition.

Supplementary tab. 4. Summary of the number of parameters of each model presented in Fig. 3 in the main text.

parents	encoder	decoder	projector
0	59,018,187	59,022,139	13,805
1	70,920,019	106,770,213	25,834
2	82,822,340	154,518,287	37,374
3	94,724,940	202,266,361	48,635
4	106,627,716	250,014,435	59,720

References

1. Kadeethum, T., Lee, S., Ballarin, F., Choo, J. & Nick, H. A locally conservative mixed finite element framework for coupled hydro-mechanical-chemical processes in heterogeneous porous media. Comput. & Geosci. 104774 (2021).

2. Kadeethum, T. et al. Non-intrusive reduced order modeling of natural convection in porous media using convolutional autoencoders: comparison with linear subspace techniques. Adv. Water Resour. 104098 (2022).

3. Kadeethum, T. et al. Reduced order modeling for flow and transport problems with barlow twins self-supervised learning. Sci. Reports **12**, 1–18 (2022).

4. Kumar, N. & DasGupta, A. On the contact problem of an inflated spherical hyperelastic membrane. Int. J. Non-Linear Mech. **57**, 130–139 (2013).

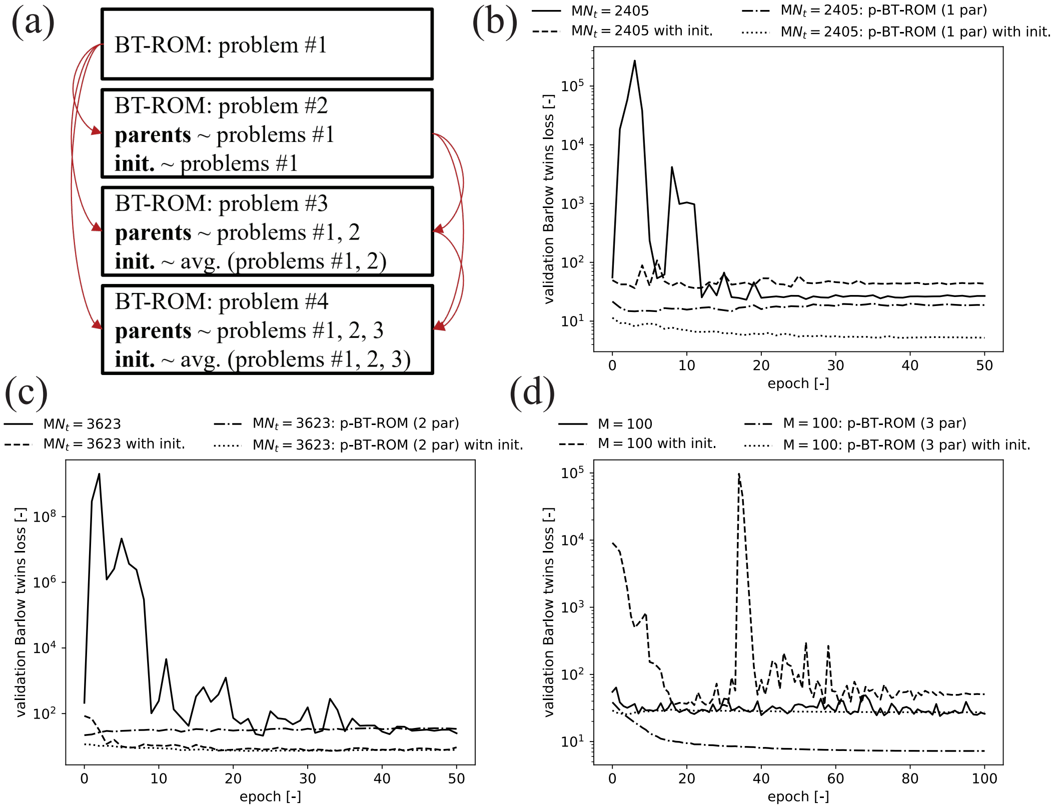

Supplementary fig. 10. Overview of p-BT-ROM Implementation and Evaluation: (a) Illustrates the p-BT-ROM framework, detailing the child-to-parent(s) relationships and the initialization process. (b) Presents the validation reconstruction loss, as defined in (Supplementary eq. 11), for Physical Problem #2 (Supplementary sec. 2.2), comparing the performance of BT-ROM, BT-ROM with initialization, p-BT-ROM, and p-BT-ROM with initialization on a small dataset ($MN_t = 2405$). (c) Shows similar validation loss results for Physical Problem #3 (Supplementary sec. 2.3), with a dataset size of $MN_t = 3623$. (d) Demonstrates the validation loss for Physical Problem #4 - A 2-Dimensional domain (Supplementary sec. 2.4) across the same ROM configurations for a dataset with $M = 100$. It is noted that Physical Problems #1 to #3 are time-dependent, whereas Problem #4 is evaluated in a steady-state condition.

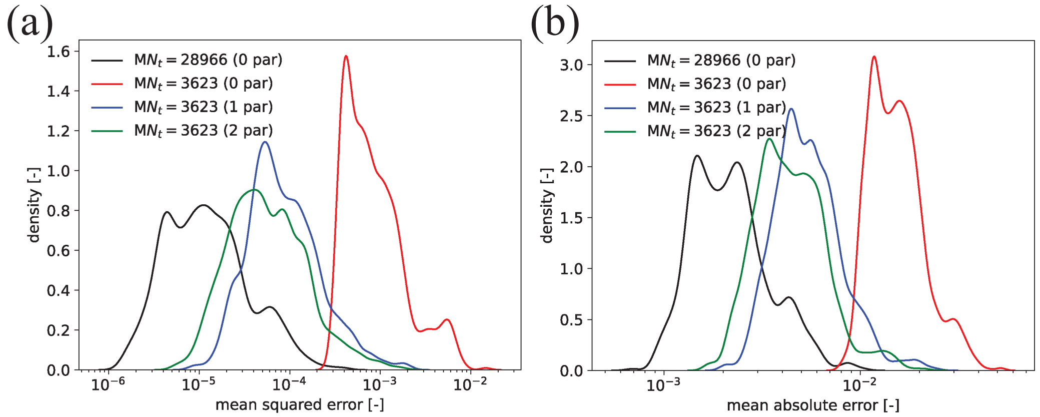

Supplementary fig. 11. Similar topology - Problem #3 (Supplementary sec. 2.3): (a) mean squared error and (b) mean absolute error results for large dataset ($MN_t = 28966$, $M = 40$) and small dataset ($MN_t = 3623, M = 5$) with a different number of parents. We note that schematic of p-BT-ROM specifies child∼parent(s) relationship is shown in Supplementary fig. 12a.

5. Luo, Y., Li, M. & Kang, Z. Topology optimization of hyperelastic structures with frictionless contact supports.

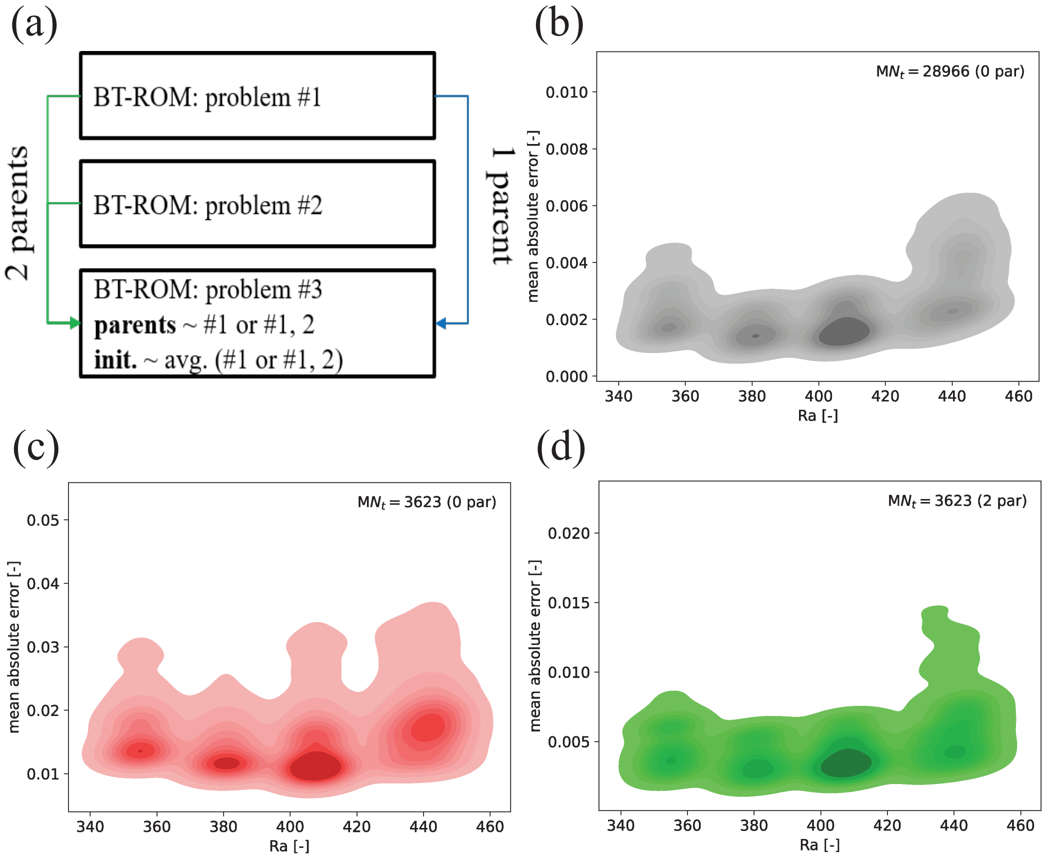

Supplementary fig. 12. Similar topology - Problem #3 (Supplementary sec. 2.3): (a) schematic of p-BT-ROM specifies child∼parent(s) relationship, mean absolute error as a function of parameter $\boldsymbol{\mu}$ results for (b) large dataset ($MN_t = 28966, M = 40$) with 0 parent, (c) small dataset ($MN_t = 3623, M = 5$) with 0 parent, and (d) small dataset ($MN_t = 3623, M = 5$) with 2 parents. We note that problems #1 to #3 are time-dependent problems, and their description can be found in (Supplementary sec. 2.1, Supplementary sec. 2.2, and Supplementary sec. 2.3).

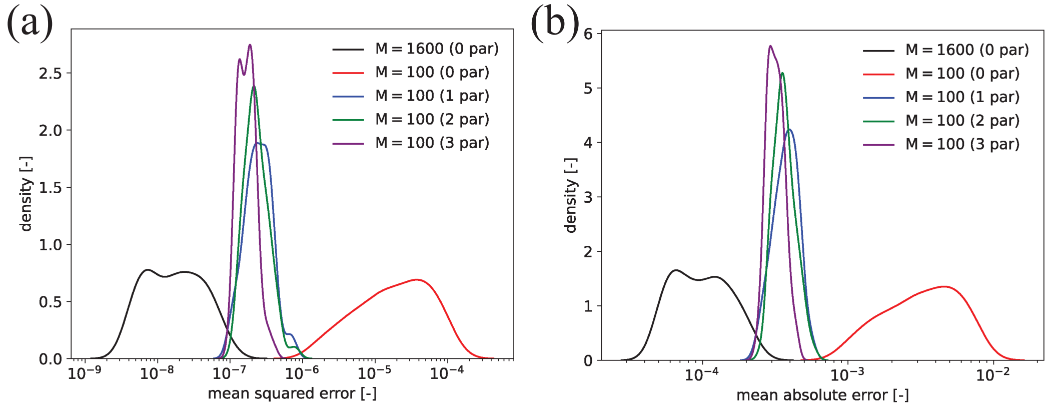

Supplementary fig. 13. Similar topology - Problem #4 (Supplementary sec. 2.4): (a) mean squared error and (b) mean absolute error results for large dataset ($M = 1600$) and small dataset ($M = 100$) with a different number of parents. We note that schematic of p-BT-ROM specifies child∼parent(s) relationship is shown in Supplementary fig. 14a.

Int. J. Solids Struct. **81**, 373–382 (2016).

6. Nezamabadi, S., Zahrouni, H. & Yvonnet, J. Solving hyperelastic material problems by asymptotic numerical method.

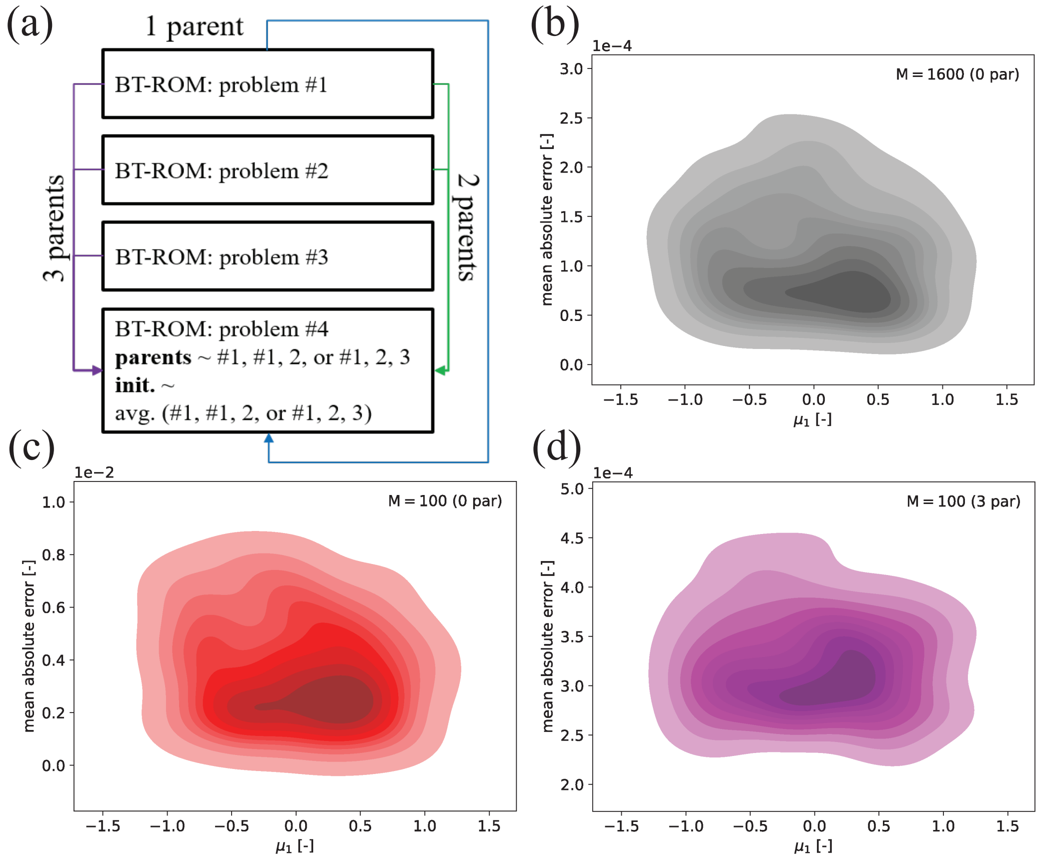

Supplementary fig. 14. Similar topology - Problem #4 (Supplementary sec. 2.4): (a) schematic of p-BT-ROM specifies child∼parent(s) relationship, mean absolute error as a function of parameter $\boldsymbol{\mu}$ results for (b) large dataset (M = 1600) with 0 parent, (c) small dataset (M = 100) with 0 parent, and (d) small dataset (M = 100) with 3 parents. We note that problems #1 to #3 are time-dependent problems while Problem #4 is at a steady-state, and their description can be found in (Supplementary sec. 2.1, Supplementary sec. 2.2, Supplementary sec. 2.3, and Supplementary sec. 2.4).

Comput. mechanics **47**, 77–92 (2011).

7. Kadeethum, T. et al. Enhancing high-fidelity nonlinear solver with reduced order model. Sci. Reports **12**, 1–15 (2022).
8. Balay, S. et al. PETSc Users Manual. Tech. Rep. ANL-95/11 - Revision 3.10, Argonne National Laboratory (2018).
9. Kadeethum, T., Jakeman, J. D., Choi, Y., Bouklas, N. & Yoon, H. Epistemic uncertainty-aware barlow twins reduced order modeling for nonlinear contact problems. IEEE Access (2023).
10. Kingma, D. & Ba, J. Adam: A method for stochastic optimization. arXiv preprint arXiv:1412.6980 (2014).
11. Loshchilov, I. & Hutter, F. Sgdr: Stochastic gradient descent with warm restarts. arXiv preprint arXiv:1608.03983 (2016).
12. Prechelt, L. Early stopping-but when? In Neural Networks: Tricks of the trade, 55–69 (Springer, 1998).
13. Prechelt, L. Automatic early stopping using cross validation: quantifying the criteria. Neural Networks **11**, 761–767 (1998).
14. Wright, G. Radial basis function interpolation: numerical and analytical developments (University of Colorado at Boulder, 2003).

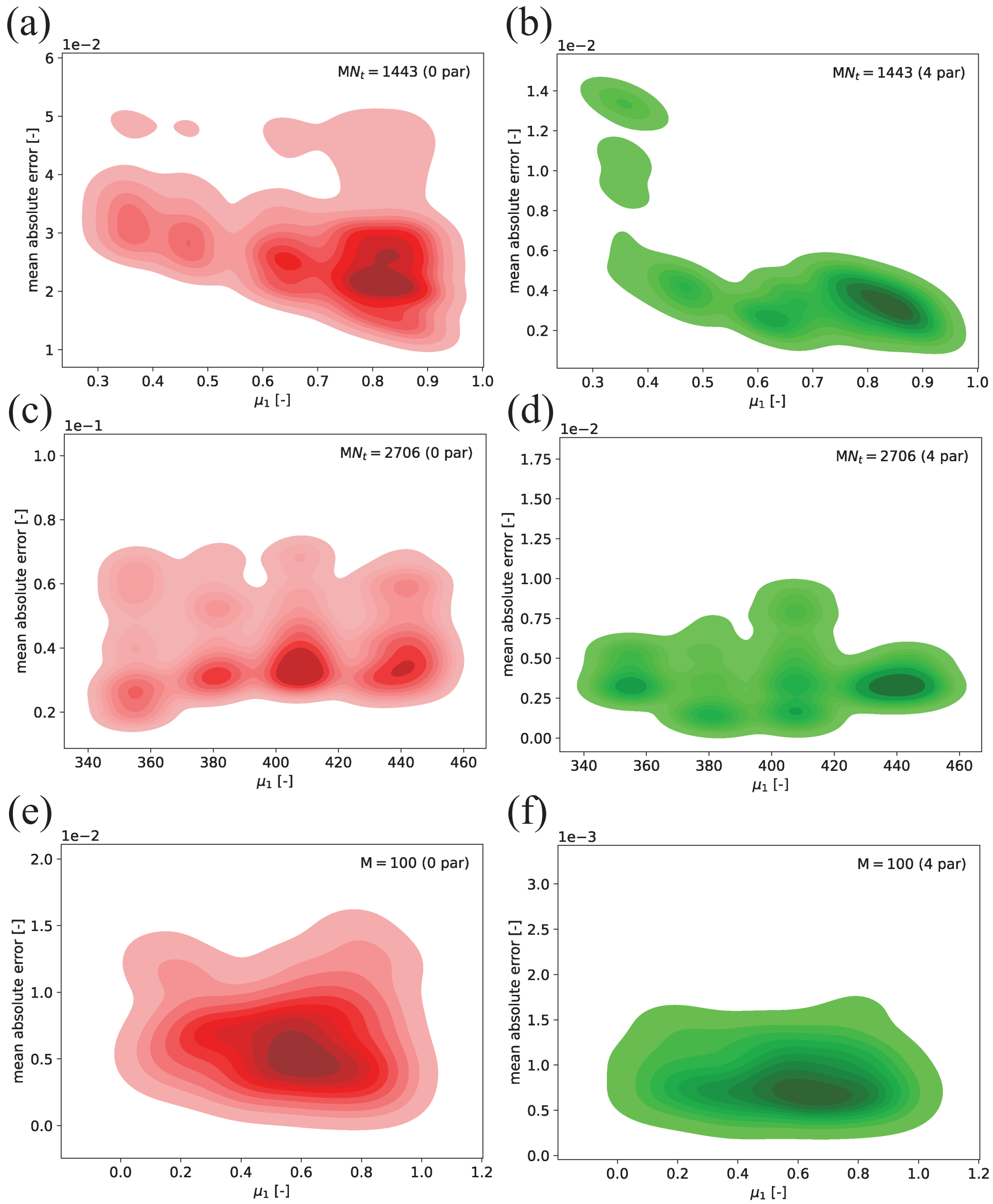

Supplementary fig. 15. Different topologies - mean absolute error as a function of parameter $\boldsymbol{\mu}$ results for (a) 0 and (b) 4 parents for Problem #2 (Supplementary sec. 2.2) using the topology shown in Supplementary fig. 1b, (c) 0 and (d) 4 parents for Problem #3 (Supplementary sec. 2.3) using the topology shown in Supplementary fig. 1c, and (e) 0 and (f) 4 parents for Problem #4 (Supplementary sec. 2.4) using the topology shown in Supplementary fig. 1d.
